# A Review of Recent Advancements in Sensor‐Integrated Medical Tools

**DOI:** 10.1002/advs.202307427

**Published:** 2024-03-09

**Authors:** Jaeho Park, Bokyung Seo, Yongrok Jeong, Inkyu Park

**Affiliations:** ^1^ Department of Mechanical Engineering Korea Advanced Institute of Science and Technology (KAIST) Daejeon 34141 South Korea; ^2^ Radioisotope Research Division Korea Atomic Energy Research Institute (KAERI) Daejeon 34057 South Korea

**Keywords:** in vivo sensing, medical tools, minimally invasive surgery, patient monitoring, sensor‐integrated medical tools, sensors

## Abstract

A medical tool is a general instrument intended for use in the prevention, diagnosis, and treatment of diseases in humans or other animals. Nowadays, sensors are widely employed in medical tools to analyze or quantify disease‐related parameters for the diagnosis and monitoring of patients’ diseases. Recent explosive advancements in sensor technologies have extended the integration and application of sensors in medical tools by providing more versatile in vivo sensing capabilities. These unique sensing capabilities, especially for medical tools for surgery or medical treatment, are getting more attention owing to the rapid growth of minimally invasive surgery. In this review, recent advancements in sensor‐integrated medical tools are presented, and their necessity, use, and examples are comprehensively introduced. Specifically, medical tools often utilized for medical surgery or treatment, for example, medical needles, catheters, robotic surgery, sutures, endoscopes, and tubes, are covered, and in‐depth discussions about the working mechanism used for each sensor‐integrated medical tool are provided.

## Graphical abstract

In this review, recent advancements in sensor‐integrated medical tools are presented, and their necessity, use, and examples are comprehensively introduced. Specifically, medical tools often utilized for medical surgery or treatment, for example, medical needles, catheters, robotic surgery, sutures, endoscopes, and tubes, are covered, and in‐depth discussions about the working mechanism of each sensor‐integrated medical tool are provided.

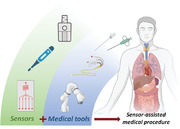

## 1 Introduction

Medicine and clinical science are continuously evolving, and this realizes a higher life expectancy with less pain and diseases. These rapid advancements in medicine and clinical sciences have been achieved by the foundation of scientific findings and technological evolutions during the last few hundred years. Historically, the scene of surgery was not something elaborate or clean, but rather bloody, dirty, and full of screaming from patients due to painful surgery.^[^ ^1^ ^]^ The first evolutionary finding and breakthrough that dramatically changed this surgery environment was anesthesia. The pain given to patients with physical and psychological burdens significantly made surgery difficult. In 1846, William T. G. Morton first utilized the anesthesia technique for surgery, which completely altered the image of surgery into a more comfortable and less painful environment.^[^ ^2^ ^]^ The invention of anesthetics accelerated the development of surgical technologies, resulting in a plethora of surgical trials. However, patients still suffered from infection during the surgery, which sometimes caused deadly results. However, this could be managed after finding the importance of hygiene and developing an antiseptic surgery technique.^[^ ^1^ ^]^ Moreover, a half meter of incision on the patient's body could be an enormous burden; thus, various approaches were tried to minimize the incision size, which could significantly reduce the pain and time during the recovery process. Among various approaches, one of the main trends is utilizing interventional medical to reach a local surgical site with a small incision. Since Werner Forssmann reported the first successful cardiovascular catheterization,^[^ ^3^ ^]^ interventional and minimally invasive surgery has been widely provided and used for safer and more accurate surgical treatments.^[^ ^4^, ^5^ ^]^ This significant trend is evolving until now, along with the rapid advancement of biosensor technology, robotics technology, augmented/virtual reality, and artificial intelligence technology.

While minimally invasive and interventional surgery is gaining popularity owing to their benefits, surgeons have to simultaneously manage multiple tiny and lengthy medical devices, such as a needle, catheter, or teleoperated robotic controller. Furthermore, controlling and monitoring all instrument‐related parameters increases surgeons' burden and can cause a momentary loss of awareness around the surgical sites.^[^ ^6^ ^]^ Therefore, providing sensation and feeling of the local surgical site to external surgeons, which is a more well‐known concept as haptic feedback, has been one of the significant issues in interventional surgery, and tremendous efforts have been made to build the medical tool system with sensory feedback by employing physical, chemical, and optical approaches. For example, mechanical properties of tissues, such as stiffness, could provide enormous information to surgeons because they could guide a differentiation between lesions and normal tissue and indicate the handling of sites.^[^ ^7^ ^]^ In the case of catheterization, as a different example, medical imaging tools commonly guide the route and insertion of the catheter. However, the catheter could easily be stuck to the blood vessel during its navigation and sometimes be misguided rather than heading to the correct surgical site, resulting in a fatal situation for the patient with several minutes of delayed treatment.^[^ ^8^ ^]^ As a result, there is an on‐going need to improve surgeon's perception during minimally invasive surgery and interventional treatments. Accordingly, various types of research about sensor‐integrated medical devices have been reported to assess physical (e.g., pressure, force, stiffness) and biochemical characteristics (e.g., pH, metabolite, protein). Initially, this was accomplished by attaching relatively bulky sensors to the ends or inside medical tools. However, thanks to the advancements in microfabrication, functional materials, and integrated sensor technologies, medical tools are increasingly incorporating miniaturized sensors, which enable sensor‐assisted medical surgery for better outcomes from the surgical procedure.

**Figure 1:**
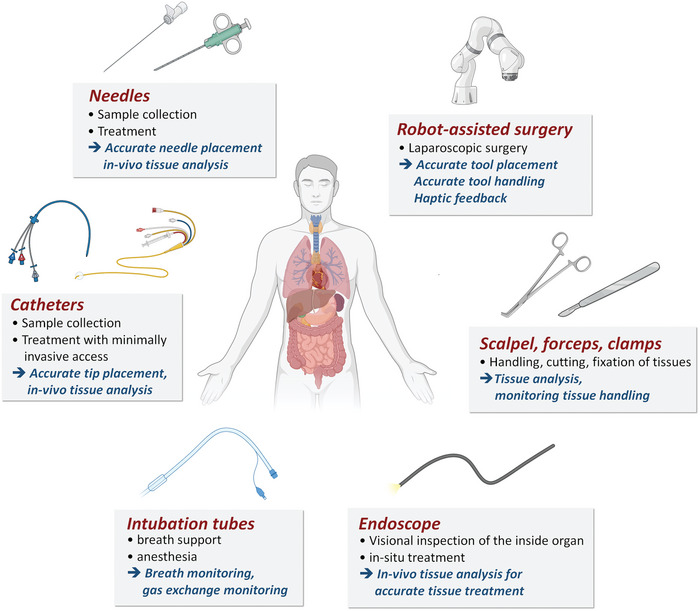
Common medical tools for minimally invasive interventional surgery and open surgery. A medical procedure is performed by a single or combination of the listed medical tools, and each of the medical tools has a distinct feature in terms of functionalities, purposes, and invasiveness. For example, needles and catheters are shaped as thin, small tubular structures to enable minimally invasive access to a specific lesion. On the other hand, a robotic surgery system and an endoscope are composed of highly functional vision and micromanipulation components to perform medical treatment through a few‐centimeter incision.

In this review article, recent advancements and examples of sensor‐integrated medical tools, mainly for, but not limited to, minimally invasive surgery, will be thoroughly introduced, and their basic working mechanism will be explained. Each medical tool is used differently with various purposes and functionalities; therefore, required parameters can vary depending on those application targets (**Figure** **1**). For example, a medical needle is commonly utilized to collect samples or specimens from inside the body while it can access the tissue or organs of interest in a minimally‐invasive manner. For this purpose, accurate positioning of the needle is the most important prerequisite for a needle‐based medical procedure; thus, monitoring force at the medical needle tip is a common strategy investigated to correctly place the needle into the target position. Meanwhile, an intubation tube, which is used to assist the patient's breathing, involves the airflow and gas exchange throughout the tube. As a result, analyzing the component of exchanged gas and its flow monitoring is commonly required. Therefore, different form factors and sensing modalities are required to incorporate the sensor system into the specific medical tools, which will be briefly explained in each section. Technically, this review article aims to explain sensor‐integrated medical tools, which could be defined as a device commonly and directly used for clinical procedures rather than stand‐alone or benchtop diagnostic devices or medical devices.

## 2 Sensor‐Integrated Medical Needle

A medical needle is one of the most utilized medical devices for various purposes, widely ranging from simple functions (e.g., blood collection, drug administration) to surgical purposes (e.g., biopsy, tissue ablation). Placement and estimation of the needle tip are essential for successful outcomes, and misguiding the needle tip leads to failed outcomes during the medical procedure and treatment, worsening the patient's prognosis. Conventionally, medical imaging tools such as ultrasound imaging, computed tomography (CT), and magnetic resonance imaging (MRI) track and guide the needle tip during minimally invasive procedures. However, the exact location of the needle tip and the boundary between different types of tissues and organs are difficult to differentiate due to the limited spatial resolution of medical imaging tools.

For this reason, various attempts have been made to integrate sensing capabilities into the medical needle for accurate needle positioning and to overcome the limitations of conventional image‐guided minimally invasive surgery. Various types of sensing modalities (e.g., electrical, mechanical, biochemical, and optical properties) have been incorporated to estimate the location of the needle tip and to differentiate types of tissue in real‐time, which will be introduced in the section.

### 2.1 Real‐Time Monitoring and Classification of Biological Tissues

#### 2.1.1 Electrical Properties for Tissue Classification

An electrical property of the biological medium has a distinct characteristic that differs from other solid‐state materials and is commonly referred to as “bioimpedance,” which specifies the electrical property of the biological medium.^[^ ^9^, ^10^ ^]^ Bioimpedance mainly originates from the cell and tissue structures.^[^ ^9^ ^]^ The cell, a fundamental component of the tissue, has a membrane to separate and protect the inner compounds of the cell from the outer environment, and this cell membrane is mainly made of a lipid bilayer.^[^ ^11^ ^]^ Because the lipid bilayer is not electrically conductive and blocks the movement of ions through the membrane, ions under an electrical field are accumulated along with the cell membrane. Therefore, the cell membrane acts as a capacitor under a static electrical field. However, under alternating electrical field conditions, these ions start to agitate rather than accumulate on the cell membrane surface, and these phenomena dominantly happen at frequencies from 100 Hz to 1 MHz. As a result, the electrical impedance of the cells and tissue is usually high at a lower frequency (<100 Hz) and low at a higher frequency (>1 MHz). Electrical impedance characteristics may vary across tissue types due to their distinct cellular components and tissue structures.^[^ ^9^, ^10^, ^12^ ^]^ Bioimpedance measurement has been researched and widely developed for biomedical and tissue analysis applications because of its straightforward measurement system and ability to provide real‐time data. Various researchers have tried to discover the electrical impedance of biological tissue, and it has been found that different types of tissues have distinct electrical impedance properties.^[^ ^13^, ^14^ ^]^ For example, in the case of cancerous tissue, their electrical impedance is usually smaller than that in normal tissues due to the different tissue structures.^[^ ^15^, ^16^, ^17^, ^18^, ^19^, ^20^ ^]^ Even among cancerous tissues, electrical impedance could vary due to the different tissue structures. Prostate cancer is a representative example of having a higher electrical impedance than normal tissue.^[^ ^15^, ^16^, ^21^ ^]^

**Figure 2:**
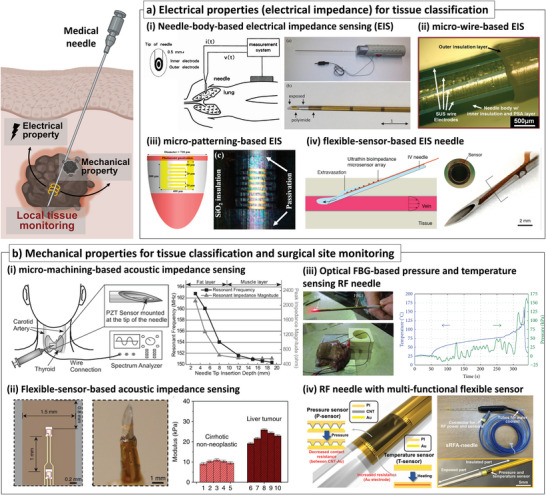
Medical needles integrated with sensors analyzing electrical and mechanical properties for tissue classification and surgical site monitoring. a) Electrical property‐based tissue classification: i) electrical impedance sensing (EIS) needle based on needle body electrode for tissue classification^[^ ^21^, ^22^ ^]^ (Reproduced with permission.^[^ ^22^ ^]^ Copyright 1994, Elsevier; Reproduced with permission.^[^ ^21^ ^]^ Copyright 2012, IEEE), ii) EIS needle with stainless steel microwires for tissue classification^[^ ^32^ ^]^ (Reproduced under the terms of the CC‐BY‐4.0.,^[^ ^32^ ^]^ Copyright 2018, Springer Nature), iii) micro‐electrode onto the hypodermic syringe needle by micro‐patterning method^[^ ^27^ ^]^ (Reproduced under the terms of the CC‐BY.,^[^ ^27^ ^]^ Copyright 2016, MDPI), iv) the sensor‐integrated needle for extravasation detection during needle insertion with flexible sensor electrodes^[^ ^36^ ^]^ (Reproduced with permission.^[^ ^36^ ^]^ Copyright 2022, Elsevier); b) sensor integration for in vivo biomechanical property analysis and surgical site monitoring: i) micro‐machining based piezoelectric sensor disc integration^[^ ^37^ ^]^ (Reproduced with permission.^[^ ^37^ ^]^ Copyright 2007, Royal Society of Chemistry), ii) micro‐patterned flexible piezoelectric sensor for in vivo analysis of vibrational characteristics^[^ ^38^ ^]^ (Reproduced with permission.^[^ ^38^ ^]^ Copyright 2018, Springer Nature), integration of iii) optical FBG^[^ ^39^ ^]^ (Reproduced under the terms of the CC‐BY.,^[^ ^39^ ^]^ Copyright 2015, MDPI) and iv) flexible pressure and temperature sensor^[^ ^40^ ^]^ (Reproduced with permission.^[^ ^40^ ^]^ Copyright 2021, Wiley) onto the RF ablation needle for local temperature and pressure monitoring during RF ablation treatment.

Numerous medical needles with electrical impedance‐detecting capabilities that can distinguish electrical properties in different tissue types have been developed for medical applications. The primary purpose of the electrical impedance sensor‐integrated medical needle is to support and estimate the location of the needle tip during needle insertion by measuring the electrical impedance and analyzing the type of tissue surrounding the needle. In the early stage of electrical impedance sensing, the coaxial structure, including the inner needle and outer guide needle, was commonly used to configure electrical impedance measurement electrodes. In 1994, a coaxial‐type needle probe with a diameter of about 0.5 mm was reported to measure the electrical impedance of living pulmonary mass in vivo.^[^ ^22^ ^]^ They successfully in vivo measured and found that the electrical impedance of normal lung tissue is significantly different from other abnormal tissue, including lung cancer, pneumonia, and metastatic tumor tissues. Meanwhile, the impedance‐based tissue discrimination system has been demonstrated,^[^ ^23^ ^]^ and the electrical properties of the local volume around the tip were measured to maximize the spatial resolution for electrical impedance measurement. However, when the measurement volume and area of the electrode decrease, an effect from ionic accumulation on the electrode surface, usually called the electrode polarization effect, arises and significantly degrades the measurement result.^[^ ^24^ ^]^ Therefore, a three‐electrode configuration was adopted instead of the conventional two‐electrode measurement, which significantly reduced the electrode polarization effect and could successfully discriminate different tissue types, such as fat and muscle. For another example, a prostate cancer biopsy process with in vivo electrical‐impedance‐based analysis was demonstrated by showing the electrical impedance of prostate cancer differs dramatically from normal prostate tissue^[^ ^15^, ^16^, ^21^ ^]^ (**Figure** **2**a(i)). The co‐axial needle structure was utilized to develop the biopsy needle with electrical impedance sensing capability. The needle body was electrically insulated using a Kapton film, and only the tip side was opened to make it electrically conductive. The sensor‐integrated needle could successfully extract prostate cancer by utilizing real‐time electrical impedance monitoring to locate the tip of the biopsy needle in the prostate tissue with great sensitivity and specificity (area under the curve (AUC) = 0.77 and 0.79 at measurement frequencies of 63.09 kHz and 251.1 kHz, respectively).^[^ ^25^ ^]^ Many researchers also tried to utilize the electrical impedance analysis to differentiate boundaries between different tissue types to locate the needle at an accurate position. In 2001, a smart needle system to confirm percutaneous kidney access was reported using electrical impedance measurement^[^ ^26^ ^]^ (Figure 2a(i)). They demonstrated the co‐axial structure‐based needle system with ex vivo porcine kidney and could successfully observe an abrupt impedance drop when the needle was inserted through kidney tissue.

The early stage of the electrical impedance sensor‐integrated medical needle utilized the needle body as an electrical conductor for impedance measurement. However, this could lead to limited electrode design and configuration for more accurate and versatile applications. Therefore, various researchers have tried to mitigate these limitations by fabricating electrodes directly on the needle surface for impedance measurement. For example, micro‐scaled electrodes could be successfully constructed on the needle surface (diameter = 0.4 mm) using traditional microfabrication techniques such as lithography and metal deposition^[^ ^27^, ^28^, ^29^ ^]^ (Figure 2a(iii)). For the pattern formation on the curved needle surface, specialized zig and pattern masks based on the bulk micromachining process were adopted.^[^ ^28^ ^]^ Based on the electrical impedance measurement, differences in electrical properties between different tissue types such as skin, fat, muscle, cancerous tissue, and intra‐abdominal cavity during laparoscopic surgery have been thoroughly investigated, which greatly ensures the usefulness of impedance‐based tissue discrimination.^[^ ^27^, ^28^, ^29^, ^30^, ^31^ ^]^ For another method, electrode formation using stainless‐steel micro‐wires for impedance measurement has been demonstrated^[^ ^32^ ^]^ (Figure 2a(ii)). Because micro‐wires were directly integrated and arranged on the surface, distinct measurement configurations were achieved, such as the multi‐point impedance sensing and 4‐electrode configuration for minimal electrode polarization effect. Moreover, the same group developed the needle integrated with electrical impedance sensing capability by utilizing flexible sensor technology for more reliable and simpler sensor integration onto the curved needle surface.^[^ ^33^, ^34^, ^35^ ^]^ Electrodes for impedance measurement were fabricated on thin polymeric substrates and then transferred to the needle surface. This approach could significantly improve the design flexibility of electrical impedance. Therefore, distinct sensing capabilities such as multi‐point impedance measurement were enabled and demonstrated.^[^ ^33^ ^]^ Most recently, a medical needle with an electrical impedance sensor array was reported to detect extravasation during needle insertion^[^ ^36^ ^]^ (Figure 2a(iv)). Extravasation is the commonly associated complication with needle insertion through the blood vessel, which can lead to local infection or edema. It was detected by applying a microfabricated flexible electrode array. The electrode surface was coated with poly(3,4‐ethylenedioxythiophate) (PEDOT) and carbon nanotubes to minimize the interfacial electrical impedance on the electrode surfaces. The feasibility of the device was further proven with animal models (mouse and pig), and the extravasation was intentionally induced by injecting the saline solution through either blood vessels or tissue. Then, extravasation was identified by observing a sudden decline in electrical impedance within the 10–100 kHz frequency range.

#### 2.1.2 Mechanical Properties for Tissue Classification and Monitoring of Surgical Site

Biological tissue has distinct mechanical properties such as stiffness, viscoelasticity, and acoustic impedance, widely ranging from soft tissues such as the brain, liver, and blood vessels to hard tissues such as tendons, bones, and teeth.^[^ ^41^, ^42^ ^]^ Therefore, tracking the mechanical force applied during medical procedures and analyzing the mechanical characteristics of the surrounding tissue can assist surgeons in needle placement by providing valuable information to surgeons. One of the representative applications is ultrasound elastography.^[^ ^43^, ^44^, ^45^ ^]^ Ultrasound elastography detects the stiffness of the biological tissue non‐invasively to analyze abnormal tissue environments such as liver fibrosis, kidney fibrosis, and prostate cancer. Likewise, several researchers have created a medical needle integrated with a mechanical sensor for real‐time tissue evaluation during medical treatment.

The mechanical stiffness of the biological tissue around the needle is often quantified by measuring its acoustic impedance, which is defined as how readily the energy of propagating acoustic or mechanical waves can be dissipated through the material. In the early stage of acoustic impedance sensors for medical needle application, a fine needle aspiration with an integrated tissue contrast sensor has been reported^[^ ^37^ ^]^ (Figure 2b(i)). A micro‐scaled lead zirconate titanate (PZT) block was used and integrated into the needle surface for tissue contrast sensing. For this, a small cavity with a diameter of 200 µm and thickness of 23 µm was formed on the needle surface using the micro electro‐discharge machining technique, and the micromachined PZT disk was mounted into the cavity. The needle integrated with a tissue contrast sensor could successfully measure the vibrational mechanical properties of different materials, including saline waters, porcine muscle, fat, and oils, demonstrating further applicability of real‐time tissue sensing during medical treatment. After that, a biopsy needle integrated with a flexible mechanical piezoelectric sensor was demonstrated^[^ ^38^ ^]^ (Figure 2b(ii)). In this report, a miniaturized actuator system for mechanical sensing was fabricated on a flexible polymer substrate; then, it was transferred onto the needle surface. This approach has significant advantages in sensor packaging because patterning and packaging brittle piezoelectric materials onto the surface with a high radius of curvature requires additional complicated fabrication steps such as micro‐machining and electrical wiring. This device demonstrated successful measurement of different Young's modulus in various types of tissues, including liver, fat, spleen, lung, and kidney. Moreover, the researchers showed the capability of differentiation between normal and tumor tissue.

In addition, undesirable mechanical forces and pressures at surgical sites often occur during minimally invasive surgery, resulting in unexpected risks to the patient. One example is the heat‐induced steam‐popping during the radiofrequency (RF) ablation treatment.^[^ ^46^ ^]^ RF ablation is the medical procedure of removing suspicious tissue parts by applying a high alternative current (AC) with an RF ablation needle inserted in the tissue.^[^ ^47^ ^]^ The flowing AC induces heating near the needle tip by agitating the ions inside the tissue, which could lead to necrosis of the surrounding tissue. However, accumulated steam due to the elevated temperature inside the tissue could induce an explosion during the ablation, which may lead to cancer recurrence. Therefore, various attempts are made to monitor the internal temperature or pressure by integrating the sensor on the RF ablation needle to avoid and control this steam‐popping phenomenon. For example, fiber‐optic sensing technologies were utilized for temperature or pressure sensing for ablation monitoring during the RF ablation procedure^[^ ^39^, ^48^ ^]^ (Figure 2b(iii)). By incorporating the optical fibers on the RF ablation needle, they could successfully measure the distribution and temporal change of pressure and temperature around the needle during the ablation procedure,^[^ ^48^ ^]^ which provides essential information for the surgeon to control ablation parameters for a safer ablation procedure. Meanwhile, flexible sensor technology has been utilized for integrating the pressure and temperature sensor onto the RF ablation needle system^[^ ^40^, ^49^ ^]^ (Figure 2b(iv)). The flexible sensor with contact‐resistance‐based pressure sensing and resistance‐change‐based temperature sensing capabilities was fabricated using nanomaterials and microfabrication techniques. This enabled a significant decrease in the sensor thickness to less than 50 µm. The RF ablation needle with temperature and pressure sensor could effectively track the change of pressure and temperature around the RF needle, and its usability was further demonstrated in pre‐clinical and clinical trials.

#### 2.1.3 Biochemical and Optical Properties for Tissue Classification

**Figure 3:**
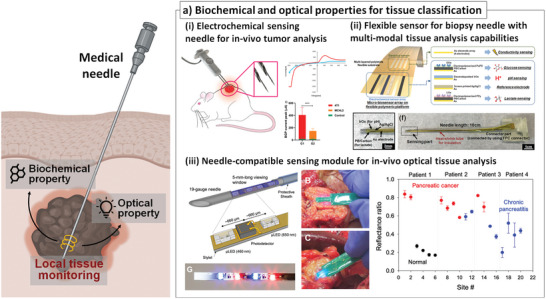
Medical needles integrated with sensors analyzing biochemical and optical properties for tissue classification. a) Electrical property‐based tissue classification: i) electrochemical sensor on the needle tip with capability of in vivo biomaterial analysis toward cancerous tissue detection^[^ ^50^ ^]^ (Reprinted with permission.^[^ ^50^ ^]^ Copyright 2020, Wiley), ii) flexible‐sensor‐based sensor integration onto the medical needle for multi‐modal biophysical/biochemical analysis^[^ ^35^ ^]^ (Reprinted with permission.^[^ ^35^ ^]^ Copyright 2020, American Chemical Society), and iii) optical‐component embedded needle stylet for in vivo optical tissue absorbance analysis for advanced tissue discrimination^[^ ^51^ ^]^ (Reproduced under the terms of the CC‐BY‐NC.,^[^ ^51^ ^]^ Copyright 2020, AAAS).

Recently, other types of sensors utilizing biochemical or optical properties of biological tissues have been tried to be integrated with the conventional medical needle for real‐time tissue analysis. For example, a needle‐shaped medical device has been reported to differentiate tissue types before/during the biopsy process by analyzing biochemical components through an electrochemical method^[^ ^50^ ^]^ (**Figure** **3**a(i)). The researchers prepared a carboxylic‐carbon‐nanotube‐coated needle tip to detect hydrogen peroxide in the microenvironment surrounding the needle. The hydrogen peroxide could indicate the cancerous tissue due to the hypoxia‐assisted glycolysis. The electrochemical sensing and analysis with a prepared needle tip could successfully differentiate and classify the types of tissues based on the mouse tumor model. In addition, similar approaches with flexible sensor technology have also been reported. The biopsy needle integrated with multi‐modal biophysical and biochemical sensors has been proposed^[^ ^34^, ^35^ ^]^ (Figure 3a(ii)). A flexible sensor with various sensing modalities such as electrical impedance, pH, glucose concentration, and lactate concentration was fabricated on a polyimide thin film using micro‐patterning techniques. The flexible sensor was further transferred and integrated onto the surface of the biopsy needle. The feasibility of multi‐modal and real‐time analysis for cancer tumor detection was demonstrated using a hydrogel phantom and ex vivo cancer‐mimicking tissues. Meanwhile, a sensor‐integrated needle employing an optoelectric device to detect pancreatic cancer was recently reported^[^ ^51^ ^]^ (Figure 3a(iii)). This system was constructed using commercially available light‐emitting diode (LED) and electronic components within a 19‐gauge medical needle and successfully discriminated the cancerous pancreatic tissue from the normal tissue in ex vivo human tissue samples.

### 2.2 Needle Placement Estimation and Visualization for Accurate Needle Positioning

**Figure 4:**
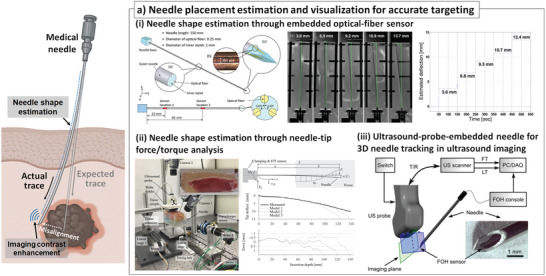
a) Sensor‐integrated medical needles for needle placement estimation and visualization for accurate targeting: needle shape estimation through i) embedded optical‐fiber^[^ ^53^ ^]^ (Reprinted with permission.^[^ ^53^ ^]^ Copyright 2010, IEEE), and ii) multi‐axial analysis of mechanical forces (normal and shear forces) at the distal end^[^ ^54^ ^]^ (Reprinted with permission.^[^ ^54^ ^]^ Copyright 2016, IEEE). iii) Imaging contrast enhancement with embedded micro‐scaled ultrasound reflection probe for 3D needle tracking in ultrasound imaging^[^ ^56^ ^]^ (Reproduced under the terms of the CC‐BY‐4.0.,^[^ ^56^ ^]^ Copyright 2017, Springer Nature).

During needle insertion in medical treatment, it is typically considered that the needle could be straightly penetrated through the patient's tissue. However, in reality, the needle undergoes various mechanical deformations, mainly bending due to significant reaction and friction forces from the tissue.^[^ ^52^ ^]^ These phenomena can be even more critical when the longer medical needle approaches a deeper site, or the medical needle with a small diameter is used. To address these limitations, several researchers have explored the use of mechanical sensors integrated into medical needles to enable real‐time shape estimation. For example, an optical fiber Bragg gratings (FBG)‐based strain sensor has been utilized and incorporated inside the needle^[^ ^53^ ^]^ (**Figure** **4**a(i)). The optical fiber has a periodic refractive index structure, and the periodic structure could reflect light with a specific wavelength. The distance between periodic structures could change when external strain is applied to the optical fiber, which changes the specific wavelength of the reflected light. Therefore, the wavelength of reflected light could be correlated or converted into applied strain along with the optical fiber. Three optical FBG sensors were radially distributed inside the needle stylet with a 120° angle interval. The wavelength change from each optical FBG was analyzed when the needle was deformed and bent. The local curvature was calculated with measured local strain at each sensing point. The needle shape and deflection could be accurately estimated using this configuration, with an error of less than 0.5 mm in deflection up to 15 mm. As a different approach, a multi‐axial force and torque sensor were used at the end of a medical needle to estimate the shape of the needle^[^ ^54^ ^]^ (Figure 4a(ii)). The sensor could monitor torque and axial force from the needle body, and the needle deflection could be recalculated with an error of roughly 1 mm to 5 mm. On the other hand, Zhang et al. integrated a strain gauge on the surface of the flexible needle.^[^ ^55^ ^]^ To estimate the multidirectional curvature deformations, strain gauges were attached at several points of the flexible needle. By reading out the strain of each strain gauge, they could successfully evaluate the deformation of the flexible needle during needle insertion.

Meanwhile, efforts to improve contrasts of medical needles in medical imaging have been also made to visualize the needle placement. For example, integrating a microscale fiber‐optic hydrophone (FOH) element into the needle tip has been reported to enhance the ultrasonic signal with information about needle location during needle placement^[^ ^56^ ^]^ (Figure 4a(iii)). The researchers attempted to obtain both a reflected ultrasound signal at the ultrasound probe and the ultrasound signal at the FOH component at the tip of the needle simultaneously. Using this information, the location of the needle tip could be reversely calculated, enabling the three dimensional (3D) location estimation and visualization of the needle tip.

## 3 Sensor‐Integrated Catheters

Catheters are hollow, thin, and flexible tubes placed into the urinary tract, bladder, and circulatory system including blood vessels and the heart.^[^ ^57^ ^]^ Catheters allow access to internal organs via natural pathways, and this can eliminate the need for open surgery and lower the risk from the procedure (e.g., tissue damage, incision size, recovery time, infection risk, and pain).^[^ ^58^ ^]^ Once installed, catheters can provide nutrients and medication or collect various biological samples (e.g., blood and urine).^[^ ^59^, ^60^ ^]^ Vascular and urinary catheters are the most common types of catheters. Vascular catheterization is utilized when non‐invasive methods (e.g., coronary CT and angiography) cannot detect cardiac lesions due to limited spatial resolution, radiation exposure, and heart rate variability.^[^ ^61^, ^62^ ^]^ Ablation is one of major applications of vascular catheters^[^ ^63^ ^]^ to treat arrhythmia cases.^[^ ^64^ ^]^ The urinary catheter is used to diagnose urinary incontinence, involuntary urine leakage, insufficient urethral closure pressure, and urinary tract infections (UI). Urinary catheters enable the drain of the bladder when patients fail to empty their bladder or to collect urine samples for analysis.^[^ ^58^, ^65^ ^]^ While catheter intervention has advantages in terms of being minimally invasive, it presents a challenge for clinicians and surgeons because the catheter must be externally navigated and monitored. Sensor‐integrated catheters can benefit minimally‐invasive surgery in various ways by providing information about the catheter placement, diagnosis (e.g., electrograms to distinguish tachycardia^[^ ^64^ ^]^), monitoring of surgical site (e.g., efficacy of tissue ablation), and postoperative tissues and complications (e.g., blockage and leakage of urethral and blood vessel^[^ ^58^, ^66^, ^67^ ^]^).

Recent developments in miniaturized and flexible sensors enable the successful integration of various sensors in the catheter, which could help in more accurate monitoring and understanding of both the internal and external environment during and after the surgery.^[^ ^58^ ^]^ This section introduces the recent advancements in sensor‐integrated catheters based on the target application of the integrated sensors in catheters such as vascular monitoring, cardiac activity and contact mapping, urinary monitoring, real‐time monitoring and assistance in surgical treatment, biomarker detection, and catheter‐induced infection.

### 3.1 Vascular Pressure Monitoring

#### 3.1.1 Resistive/Piezoresistive Sensors for Blood Pressure Measurement

Traditionally, arterial blood pressure was measured by various devices, such as a fluid‐filled catheter connected to a mercury manometer by Poiseuille (1828) and a kymograph by Ludwig (1848).^[^ ^75^ ^]^ Afterward, metallic strain sensors such as metal wire bonded to a prismatic bar were used to measure intra‐arterial pressure.^[^ ^76^ ^]^ After strain gauges based on germanium and silicon were reported to have higher gauge factors than metal strain gauges, the silicon‐based strain gauge has been widely utilized for pressure measurement.^[^ ^77^, ^78^ ^]^ For example, a silicon strain gauge on a rubber diaphragm was reported, and further vascular pressure measurement was demonstrated in the aorta of a dog.^[^ ^79^ ^]^

**Figure 5:**
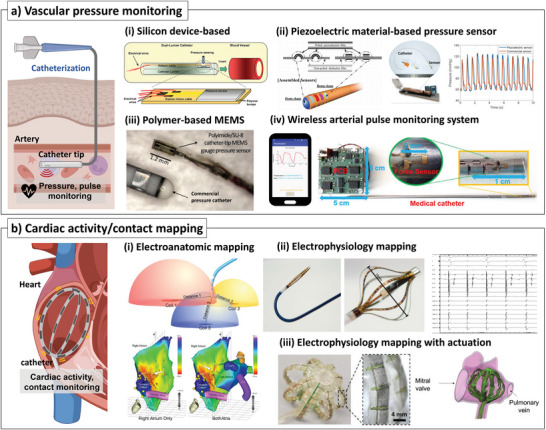
Sensor‐integrated catheters for vascular pressure monitoring and cardiac activity/contact mapping. a) Vascular pressure monitoring: i) MEMS fabricated sensor‐integrated piezoresistive pressure sensor mounted into the dual lumen neonatal catheter^[^ ^68^ ^]^ (Reproduced with permission.^[^ ^68^ ^]^ Copyright 2004, IEEE), ii) a flexible and biocompatible piezoelectric force sensor made of PVDF‐TrFE film mounted on the catheter^[^ ^69^ ^]^ (left figure) (Reproduced with permission.^[^ ^69^ ^]^ Copyright 2012, IEEE); and piezoelectric sensor with BaTiO_3_ filler in the PDMS matrix integrated into the catheter^[^ ^92^ ^]^ (right figure)(Reproduced with permission.,^[^ ^92^ ^]^ Copyright 2021, Wiley), iii) polymer substrate‐based MEMS pressure piezoresistive sensors on catheter tip^[^ ^70^ ^]^ (Reproduced under the terms of the CC‐BY‐2.0 license.,^[^ ^70^ ^]^ Copyright 2012, Springer Nature), iv) real‐time arterial pulse monitoring with near‐field communication (NFC) from sensor‐integrated catheter^[^ ^71^ ^]^ (Reproduced with permission.^[^ ^71^ ^]^ Copyright 2022, American Chemical Society). b) Cardiac activity/contact mapping: i) illustration representing the electro‐anatomic mapping using CARTO (Biosense Webster, United States) (top figure) and generated 3D cardiac chamber anatomic structure (bottom figure) in combination with electrophysiology mapping catheter (NaviSTAR, Biosense Webster, United States)^[^ ^72^ ^]^ (Reproduced under the terms of Creative Commons Attribution License.^[^ ^72^ ^]^ Copyright 2008, Elsevier), ii) electrophysiology mapping with basket‐shaped catheter (left figure) and pulmonary vein potentials captured from leads on basket‐shaped catheter (right figure)^[^ ^104^ ^]^ (Reproduced with permission.^[^ ^104^ ^]^ Copyright 2015, Heart Rhythm Society and Elsevier), iii) soft robotic sensor arrays for conformal contact, an actuatable multilayered sensor for atrial mapping performing electrophysiology mapping with actuation^[^ ^74^ ^]^ (Reproduced under the terms of CC BY‐NC license.,^[^ ^74^ ^]^ Copyright 2008, AAAS).

With the advancement of the silicon process and micro‐machining technology,^[^ ^80^ ^]^ micro‐electromechanical systems (MEMS) piezoresistors are manufactured in a miniaturized form, making them suitable for cardiovascular pressure measurement.^[^ ^81^ ^]^ The piezoresistive sensor made with polysilicon with µm dimension for catheter application could measure −20 to 300 mmHg with a sensitivity of 2 µV V^−1^ mmHg^−1^.^[^ ^82^ ^]^ The authors performed the blood pressure measurements in coronary arteries for balloon angioplasty and found reduced pressure in the aorta with different stenosis severity. However, the sensor showed unstable performance depending on temperature changes. As another attempt, a MEMS‐based pressure sensor was placed into the dual‐lumen neonatal catheter (**Figure** **5**a(i)) with an outer diameter of 1.67 mm.^[^ ^68^ ^]^ With flip‐chip bonding, commercial pressure sensors were mounted on the silicon substrate. The MEMS pressure sensor could measure the pressure range of 80–120 mmHg inside the pressure chamber. However, it suffered thermal drift, a common issue of silicon piezoresistor by high leakage current.^[^ ^83^ ^]^ One of the alternatives to resolve this issue is the single‐element piezoresistor (SEP), which could significantly reduce its size while improving its sensitivity by eliminating the requirement of a Wheatstone bridge for accurate measurement.^[^ ^84^ ^]^ Thus, with more optimization of SEP, such as length, width, junction depth, output terminal shape, and doping concentration, the sensor‐integrated catheter for neonatal intensive care application was successfully demonstrated.^[^ ^85^, ^86^ ^]^ However, Si piezoresistors, being rigid, pose a challenge for attaching them onto a curved catheter surface. Until now, MEMS pressure sensors and technologies for miniaturized packaging have led to successful commercialization from several companies (e.g., Amphenol, TEconnectivity, Millar) and utilization in clinical fields, which are used for minimally invasive and interventional pressure measurement.

Sensors fabricated on a polymer substrate must have conformal contact on the inner/outer surface of the catheter to acquire a better sensor output signal with durability. For example, MEMS gauge pressure sensors (Figure 5a(iii)) were microfabricated on a polyimide substrate and adhered to a flexible catheter tip.^[^ ^70^ ^]^ Gold gauge patterns were fabricated on a polyimide surface, and a µm‐sized SU‐8 pressure chamber was mounted for pressure transduction by changing its volumes under external pressure. Additional microfabricated gold patterns were fabricated as a resistive temperature detector for temperature compensation, while the strain gauge was used for pressure measurement. An in vivo experiment with a mouse model showed that the sensor mounted on the catheter could measure heart rate and carotid blood pressure in real‐time.

#### 3.1.2 Piezoelectric Sensors for Blood Pressure Measurement

Piezoelectric materials generate electric signals in response to applying strain.^[^ ^87^ ^]^ Piezoelectric sensors can monitor the low pressure of interest (5 kPa)^[^ ^88^ ^]^ and have high sensitivity in dynamic pressure, fast response, and a simple structure compared to other pressure/force sensors.^[^ ^89^, ^90^ ^]^

The pressure sensors for integration with the catheter require a sufficiently thin size, flexibility, and biocompatibility. Therefore, poly(vinylidene fluoride)‐*co*‐trifluoroethylene (PVDF‐TrFE) is gaining more attention because of its flexibility and biocompatibility, unlike other piezoelectric materials, such as BaTiO3 ceramics. Furthermore, the PVDF‐TrFE piezoelectric thin film can be manufactured on a MEMS‐fabricated mold, making it appropriate for mass‐producing devices and allowing the creation of 3D morphology of PVDF‐TrFE film in µm dimension. For example, a PVDF‐TrFE‐based force sensor and its catheter application (Figure 5a(ii)) have been reported.^[^ ^69^ ^]^ The film was made into a dome and bumped shapes with spin‐coating on MEMS fabricated mold and attached to a catheter surface. The dome and bump shapes correspond to the proximal end and surface of the catheter, respectively. A localized polling technique was employed to alleviate sensor crosstalk whereby only the defined region of the film surface, comprising the dome and bump areas, was polarized, while the remaining areas were left undisturbed. The sensor could monitor applied forces as low as 40 and 25 mN from bump‐shaped and dome‐shaped sensors, respectively, which is a suitable force range for potential use for tactile sensing during surgery.

As another example, the catheter with a PVDF‐TrFE‐based pressure sensor was demonstrated to evaluate blood pressure wirelessly during trauma care surgery. A PVDF‐TrFE thin film was microfabricated by ion etch, dry etch, and lift‐off processes and placed on a Kapton film to be integrated on either side of the catheter balloon.^[^ ^91^ ^]^ In a 3D vascular model, the sensor measured blood pressure with a sensitivity of 99 V mmHg^−1^, higher than that of a commercial pressure sensor (25.3 V mmHg^−1^). In addition, real‐time arterial pulse monitoring was conducted with a piezoelectric‐sensor‐integrated catheter (Figure 5a(iv)).^[^ ^71^ ^]^ PVDF‐TrFE piezoelectric material was sandwiched between Au metallic electrodes and put onto a medical catheter. The sensor measured pressure lower than 40 kPa with a sensitivity of 0.63 V Pa^−1^. Wireless transmission of arterial pulse monitoring was successfully demonstrated with a connection of a near‐field communication circuit. The sensor showed stable operation under more than 400 000 cycles of loadings. The sensor was integrated into a catheter and tested in a synthetic anatomic phantom, which shows tracking of mechanical actuation. Also, the catheter tip in contact with the skin above the carotid artery at the neck showed an arterial pulse signal successfully.

While PVDF and PVDF‐TrFE exhibit desirable features for catheter applications, such as sensitivity and flexibility,^[^ ^90^ ^]^ there is still scope for further enhancement. The sensitivity, piezoelectricity, and flexibility of the piezoelectric devices can be improved by embedding inorganic particles into a flexible polymer matrix. For example, a piezoelectric sensor made by merging BaTiO_3_ filler in the polydimethylsiloxane (PDMS) matrix was developed and integrated into the catheter for measuring stenosis (Figure 5a(ii)),^[^ ^92^ ^]^ which is an obstruction of flow and pressure loss that can lead to ischemia.^[^ ^93^ ^]^ Blood pressure has been established as an indicator of stenosis,^[^ ^94^ ^]^ which can be further quantified by fractional flow reserve (FFR). FFR is calculated as the ratio between maximum flow with and without stenosis. The researchers placed a BaTiO_3_/PDMS nanocomposite on the distal end of the guide wire and tested it in a phantom arm that simulated cardiac functions. The sensor successfully measured the blood pressure in the phantom arm, demonstrating a sensitivity of ≈0.75 mV kPa^−1^, equivalent to 100 µV mmHg^−1^. This level of sensitivity is comparable to other catheters integrated with sensors.^[^ ^91^, ^95^ ^]^

Despite the benefits of piezoelectric sensors mentioned earlier, several limitations remain. The signal strength of the sensor is heavily reliant on the size of the piezoelectric device, meaning that miniaturizing the sensor is challenging due to the reduced signal output associated with smaller dimensions. Moreover, the polling process is required to induce effective piezoelectricity, which requires more equipment and fabrication processes.^[^ ^96^, ^97^ ^]^ The sensors were capable of continuous pressure measurement within a dynamic chamber of a pressure chamber and could also be utilized on a catheter balloon.

### 3.2 Cardiac Activity/Contact Mapping

Electroanatomic mapping, also categorized in cardiac mapping technology, produces an anatomic structure map and correlates electrophysiology with cardiac location.^[^ ^72^ ^]^ It is essential for effective ablation because it helps understand the morphology of heart chambers during minimally invasive procedures such as RF ablation. CARTO (Biosense Webster Inc., CA, USA) and EnSite NavX (Abbott Laboratories, IL, USA) are examples of commercially available electroanatomic mapping systems for heart chambers. These systems utilize different techniques such as magnetic mapping (CARTO) and electrical impedance‐based (EnSite NavX).^[^ ^98^, ^99^ ^]^ Magnetic‐based mapping in CARTO detects the externally applied magnetic field with catheter electrodes; similarly, impedance‐based mapping in EnSite NavX detects the externally applied electric field with catheter electrodes (Figure 5b(i))^[^ ^72^ ^]^ Nevertheless, there are drawbacks to electroanatomic mapping such as dislocation‐related mapping shifts and the requirements for X‐ray fluoroscopy for catheter navigation.^[^ ^58^, ^99^ ^]^ Although dislocations between images can be improved by fusing MRI/CT images,^[^ ^99^ ^]^ mismatch cannot be completely eliminated,^[^ ^100^ ^]^ leaving room for potential research. For example, electroanatomic and electrophysiology mapping are often combined to overcome these challenges, which can compensate for each method's limitations.

Electrophysiology mapping records electric signals from multiple sites in the heart to diagnose the arrhythmia and to pinpoint the exact location where the abnormal signal is occurring.^[^ ^64^ ^]^ However, insufficient contact with the catheter probe during the electrophysiology mapping generates an incorrect surface profile, leading to inaccurate electrical characteristics of a tumor.^[^ ^101^ ^]^ For example, A basket‐shaped catheter^[^ ^73^, ^104^ ^]^ is one of the techniques used to perform electrophysiology mapping with improved contact (Figure 5b(ii)). This catheter is designed with eight flexible nitinol splines that can be expanded from the guiding sheath and conform to the shape of the heart chamber. Every spline has wires with multiple electrodes that determine the electrode‐tissue contact by detecting ventricular capture. As another benefit, multiple measurement points on catheter splines can acquire multiple profile information with a single trial and reduce repetitive processes compared to single‐point measurement approaches.^[^ ^102^, ^103^ ^]^ The multielectrode basket‐shaped catheter can detect ventricular tachycardia (VT) in pigs defined by tachycardia (overdriving pacing) measured from all electrodes. The device has been further developed commercially and proved its efficacy in clinical studies.^[^ ^104^, ^105^, ^106^ ^]^

In addition, to provide exact information on the contact profile, soft robotic actuators were employed in a basket‐shaped catheter (Figure 5b(iii)).^[^ ^74^ ^]^ An actuatable multilayered sensor was fabricated for atrial mapping by utilizing heat pressing, laser cutting, and flexible PCB‐based electrical sensors for voltage mapping. With the hydraulic operation, the sensor array showed over 85% conformability when simulated in a 3D‐printed left arterial model made of soft materials.

### 3.3 Real‐Time Monitoring and Assistance in Surgical Treatment

**Figure 6:**
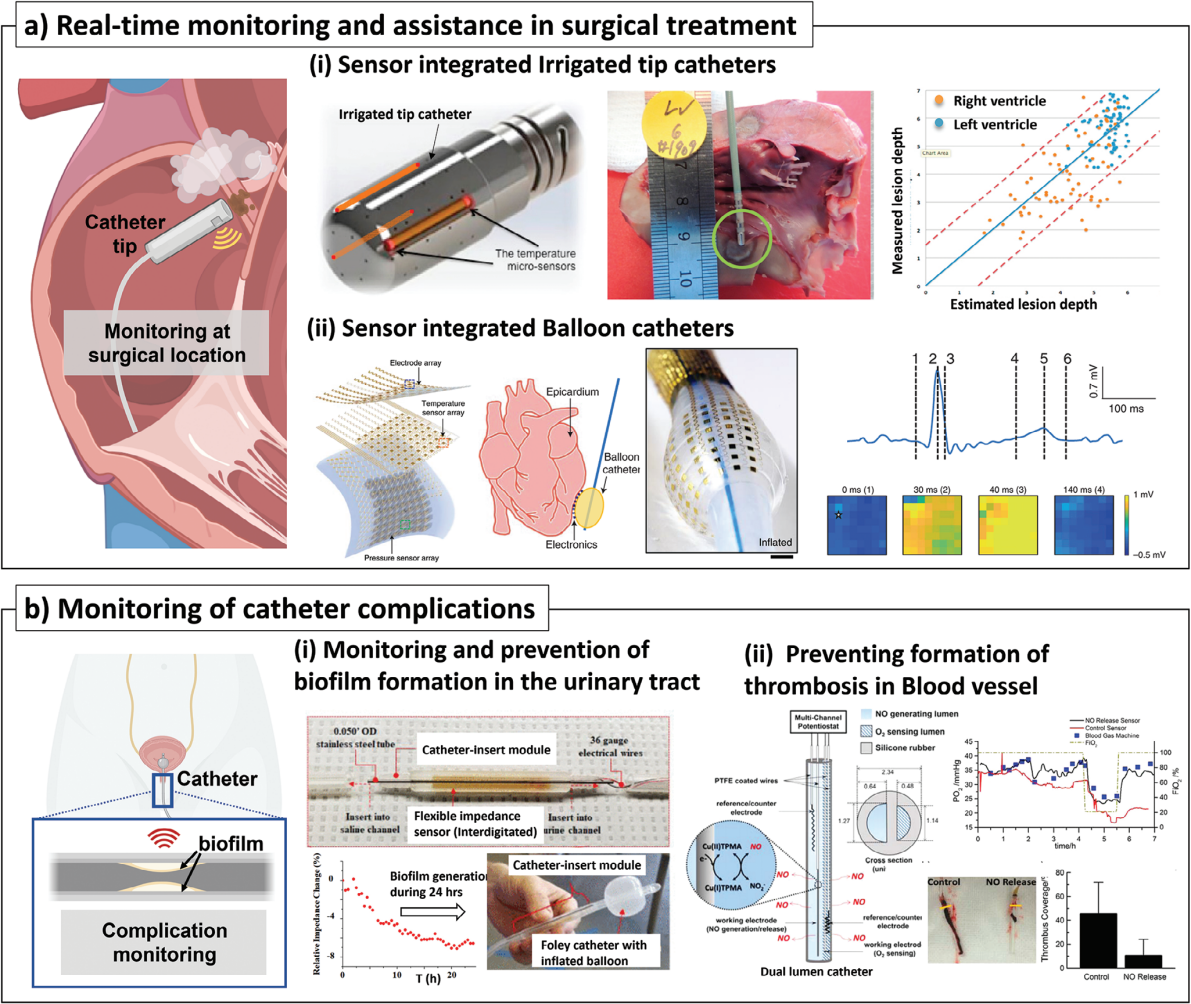
Sensor‐integrated catheters for real‐time monitoring and assistance in surgical treatment, and the monitoring of catheter complications. a) Real‐time monitoring and assistance in surgical treatment: i) lesion formation prediction with thermocouple‐embedded irrigated tip (left figure), left ventricles of swine with RF ablated lesions were dissected (middle‐figure), and correlation between estimated and actual lesion depth (right figure)^[^ ^107^ ^]^ (Reprinted with permission.^[^ ^107^ ^]^ Copyright 2016, Elsevier Inc.), ii) multi‐functional catheter with soft sensor array for RF ablation and IRE^[^ ^108^ ^]^ (Reprinted with permission.^[^ ^108^ ^]^ Copyright 2020, The Author(s), Springer Nature Limited). b) Monitoring of catheter complications: i) sensor‐integrated catheter for monitoring and prevention of biofilm formation in the urinary.^[^ ^109^ ^]^ (Reprinted with permission.^[^ ^109^ ^]^ Copyright 2021, IEEE), ii) preventing the formation of thrombosis in blood vessels utilizing dual lumen catheter for NO generation and partial oxygen pressure measurement.^[^ ^130^ ^]^ (Reprinted with permission.^[^ ^130^ ^]^ Copyright 2015, American Chemical Society).

RF ablation is the medical treatment to eliminate abnormal biological tissues by generating heat through applied alternating current (AC).^[^ ^111^ ^]^ One application of RF ablation is to treat arrhythmia, and arterial fibrillation characterized by chaotic and uncoordinated atrium contraction.^[^ ^100^ ^]^ The ablation depth during RF ablation should be accurately monitored to achieve better outcomes and prevent potential complications such as excessive heating, steam popping,^[^ ^112^ ^]^ and thrombosis. For ablated depth estimation, a catheter with irrigation and temperature‐sensing functions has been developed.^[^ ^113^ ^]^ This catheter combined a thermocouple in the irrigated tip allowing for precise temperature readings at the catheter tip when it contacts with the ablated tissue. In addition, the irrigated‐tip catheter also contained function for a contact force mapping using three optical fibers in three directions. The in vivo results in a canine thigh showed a correlation between increased contact force during RF ablation, which can be further utilized to detect steam popping and thrombus. However, the researcher noted that the study was limited because the demonstration was performed in a canine thigh instead of a beating heart. Another group^[^ ^107^ ^]^ (**Figure** **6**a(i)) established a model for estimating ablation depth during RF ablation. Ablation parameters (e.g., impedance drop, temperature, ablation duration, contact force, and orientation factor) recorded from a commercial irrigated‐tip catheter and an electroanatomic mapping system (CARTO) were utilized for estimation. The efficacy of the prediction model was assessed with the swine model, and the results showed a correlation between the estimated and measured ablation depth. The difference between the estimated and actual ablation depth was 1.5 mm in 91.6% of samples.

Recent advances in soft electronics have enabled to mounting of multi‐functional sensors on the soft and round surface of balloon catheters. For example, physiological parameters were measured with a contact force mapping electrode on a balloon catheter to verify ablation results.^[^ ^114^ ^]^ These sensors were fabricated with materials that can merge directly with the thin elastic membrane of the catheter balloon with RF ablation electrodes. The sensor array can measure impedance, detect location, and verify mechanical contact with heart tissue, which enables to obtaining numerous information during the RF ablation procedure such as temperature, flow, tactile, and electrophysiological data. Similarly, flow monitoring in a flexible balloon catheter is useful for detecting vascular blockage.^[^ ^115^ ^]^ The flow was measured by monitoring the temperature drop near the tip of the balloon catheter with a heating element and thermistor. In the report, various parameters (e.g., blood flow, electrical impedance, contact) were monitored in vitro with chicken breast. In addition, its usefulness was verified in an in vivo animal study, and increased blood flow was observed with an epinephrine injection.

In addition to RF ablation, irreversible electroporation (IRE) is another technique used in medical procedures. A microsecond electrical pulse is applied to cells inducing cell death with defects in lipid bilayers.^[^ ^116^, ^117^ ^]^ In contrast to the RF ablation, the nonthermal nature of IRE can preserve the extracellular matrix, hence the damages in neighboring tissues can be minimized.^[^ ^118^ ^]^ A sensor‐integrated flexible ablation catheter for both RF ablation and IRE was reported, and multi‐parametric sensing (e.g., contact mapping, temperature monitoring, and physiological testing capabilities) was implemented (Figure 6a(ii)).^[^ ^108^ ^]^ The catheter was composed of multiple components including a multi‐layered sensor and an actuation part. The multimodal strategy was first verified with an ex vivo Langendorf rabbit model. An electrode array recorded electrograms and voltage maps from a rabbit heart showing the pulse wave on the heart site. In an ex vivo rabbit model, heart rhythm could be restored after IRE in the arrhythmia model. In addition, RF ablation was performed in conjunction with mapping the temperature. Electrograms could measure ventricular excitation, repolarization, and ablation on a human cardiac surface ex vivo, which could be verified on an immunohistogram.

### 3.4 Monitoring of Catheter Complications

Despite their advantages, catheters can cause complications such as infections and thrombosis, notably catheter‐associated UI^[^ ^119^, ^120^ ^]^ and catheter‐related bloodstream infections.^[^ ^121^ ^]^ When catheters are inserted, the environment around the catheter can be conducive to bacterial growth,^[^ ^122^ ^]^ which leads to the pathogenesis of infection.^[^ ^123^ ^]^ To deal with catheter‐associated UI, a study on a bioelectric effect^[^ ^124^ ^]^ was utilized for bacterial detection and biofilm treatment function on indwelling catheters (Figure 6b(i)).^[^ ^109^ ^]^ The impedance values with interdigitated electrodes on the catheter tip were monitored to identify biofilm formation. This monitoring and treatment process was remotely controlled with wireless communication technologies. In addition to this, The Foley catheter with a flexible capacitive sensor has been reported to diagnose a urinary infection.^[^ ^125^ ^]^ A capacitive sensor is a sensor that can monitor the parameter of interest through changes in the capacitance of the capacitor, which is usually composed of two or more conductors with interfacial dielectric materials.^[^ ^126^ ^]^ The capacitance array with a PDMS spacer was fabricated with nine sensing points, and the device was mounted on the 7 French gauge) (outer diameter = 2.333 mm) urethral catheter. The capacitance of the sensor showed capacitance changes with external pressure from 0.36 to 0.64 fF cm^−1^ in the air pressure chamber. To minimize the effect of a parasitic capacitance (*C*_p_) in the device, an additional electrode was placed as a reference, unaffected by the externally applied pressure. The *C*_p_ can be compensated with estimated capacitance between the sensor and auxiliary reference electrode. Moreover, the *C*_p_ caused by the fringe effect on the edge of the capacitor can significantly distort the calibration of the sensor.^[^ ^127^ ^]^ As a potential solution, a supercapacitor with high dielectric constant materials can considerably enhance the performance by improving sensitivity and value of capacitance, which relatively minimizes the effect of *C*_p_. For example, the urinary catheter with a supercapacitor pressure sensor array was reported to determine the presence of urinary infection.^[^ ^67^ ^]^ The catheter had five distributed supercapacitor pressure sensors composed of an electrode and paper‐based electrolyte. It can obtain the pressure distribution for monitoring urinary infections with a high sensitivity of 30–50 nF N^−1^ with minimal effect from the external aqueous environment. An in vitro test was performed on extracted sheep bladder and urethra with applying pressure, which showed the sensor response corresponding to applied external force with negligible drift. In addition, a deformable capacitive pressure sensor on an inflatable balloon of a Foley catheter was proposed for continuous monitoring of intra‐abdominal pressure for early intervention for abdominal compartment syndrome and intra‐abdominal hypertension.^[^ ^128^ ^]^ The capacitive pressure sensor was fabricated by spin‐casted Ecoflex and cyanoethyl pullulan on an abrasive paper to form a micro‐structured dielectric layer. For sensor electrodes, vacuum‐filtrated AgNW layers were utilized. The pressure sensors were integrated into a flexible sleeve for measuring pressure, which could be stretched and attached to a Foley catheter.

Blood clots produced on the surface of a sensor‐integrated intravascular catheter can affect catheter‐related bloodstream infections and the risk of thrombosis in patients. To address blood clots, the catheter balloon incorporated with a capacitive sensor was reported to monitor the blood vessel stiffness and occurrence of thrombosis.^[^ ^129^ ^]^ An active *C*_p_ cancellation technique was employed to compensate for *C*_p_. This active cancellation improved a resolution to 16 aF and a signal‐to‐noise ratio (SNR) of 53.8 dB, 19.6 dB higher than the measurement without Cp cancellation. Another example is a sensor‐integrated catheter that can actively prevent blood clots by monitoring in vivo partial oxygen pressure (pO_2_) and generation of nitric oxide (NO).^[^ ^130^ ^]^ Intravascular sensor applications can be challenging due to potential complications such as thrombosis formation. NO generation can significantly improve the stability of intravascular sensors^[^ ^131^ ^]^ by reducing infection risk from bacteria.^[^ ^110^, ^132^ ^]^ One lumen was filled with nitrite and copper (II)‐tri(2‐pyridylmethyl)amine (CuTPMA) catalyst for the generation and release of NO (Figure 6b(ii)).^[^ ^130^ ^]^ The other lumen was with a fluoropolymer‐coated Pt working electrode and an silver/silver chloride (Ag/AgCl) reference electrode for monitoring oxygen reduction. The blood pO_2_ concentration was monitored by reading a current profile generated from the oxygen reduction. The device was tested in pig arteries in vivo with and without generation. As a result, it was shown that the released NO significantly prevented the formation of blood coagulation around the oxygen sensor, which led to more stable and reliable oxygen measurement.

### 3.5 Biomarker Detection

**Figure 7:**
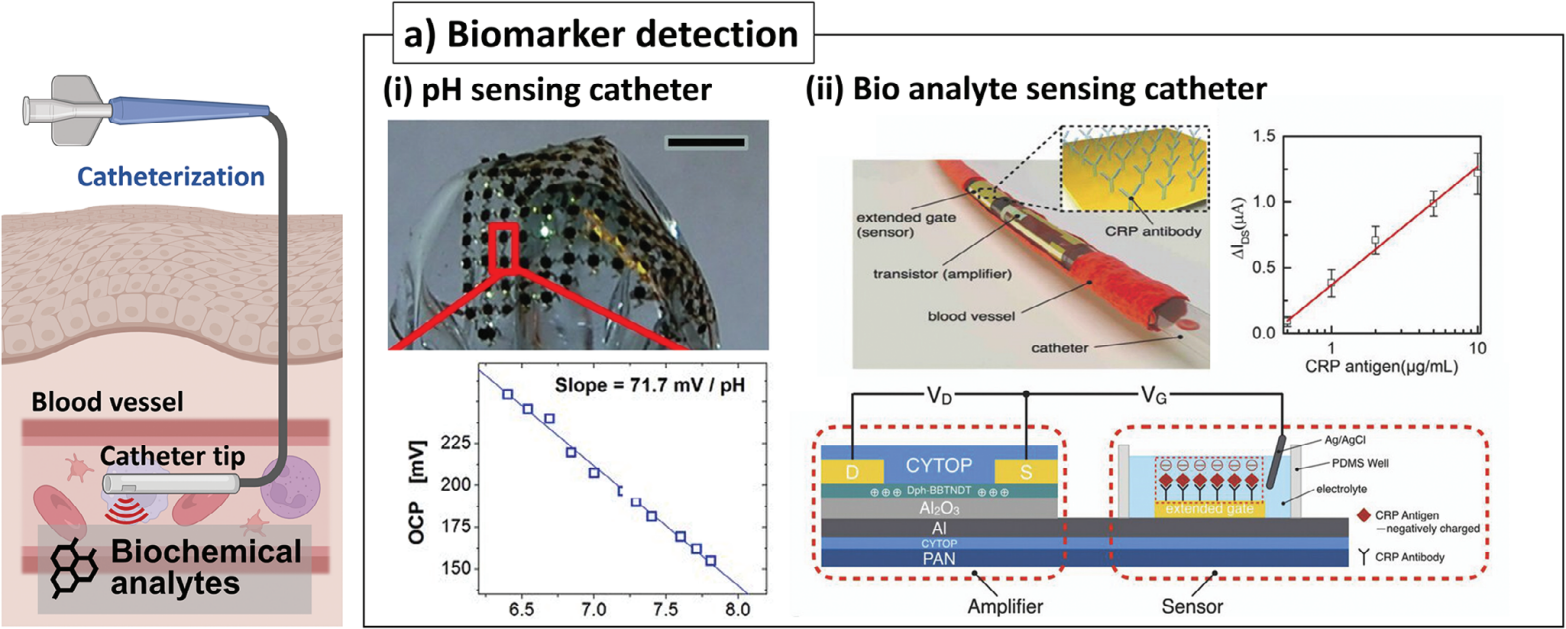
Sensor‐integrated catheters for the biomarker detection. a) Biomarker detection: i) flexible pH sensor array with electroplated IrOx layer on the balloon catheter^[^ ^134^ ^]^ (Reproduced with permission.^[^ ^134^ ^]^ Copyright 2014, Wiley‐VCH) and ii) a vascular catheter with OFET‐based sensors for CRP detection^[^ ^137^ ^]^ (Reproduced under the terms of the CC BY license,^[^ ^137^ ^]^ Copyright 2018, Wiley).

Monitoring of cardiac pH in clinics is necessary since fluctuating heart pH can result from various cardiovascular diseases such as ischemic symptoms,^[^ ^133^ ^]^ therefore there have been attempts to develop pH sensing in a catheter. For example, a balloon catheter integrated with elastic pH sensor arrays was developed (**Figure** **7**a(i)).^[^ ^134^ ^]^ An iridium oxide (IrOx) is used as a pH sensor because of its excellent stability in the physiological environment and super‐Nernstian response with a sensitivity of around −51 mV pH^−1^.^[^ ^135^ ^]^ In this work, the interconnected IrOx sensors were fabricated on a low elastic modulus silicone substrate and the sensor array was conformally adhered to the tissue with large deformation. Its usefulness was fully demonstrated in ex vivo studies with rabbit hearts, and a human heart to detect the ischemic human heart through the real‐time monitoring of pH distribution.

C‐reactive protein (CRP) is an inflammation biomarker circulating in the bloodstream, and CRP levels in the serum rapidly increase in response to inflammation.^[^ ^136^ ^]^ A vascular catheter with an antibody‐based CRP sensor (Figure 7a(ii)) was implemented for CRP sensing.^[^ ^137^ ^]^ An organic field‐effect transistor(OFET) was utilized to take advantage of the flexibility, making the sensor excellent for conformal contact with medical equipment. The thickness of the OFET device is around 630 nm, enabling flexibility under extreme bending situations (bending radius of 1.5 µm). The transferred OFET showed minor changes in its characteristics (e.g., threshold voltage, carrier mobility, and dielectric capacitance). Furthermore, the device encapsulated with fluoropolymer can be sterilized in the autoclave for more than 30 min, rendering it suitable for real medical applications. The OFET‐based CRP sensor demonstrated a sensitivity of 1 µg mL^−1^ (Figure 7a(ii)) and exhibited high selectivity to CRP with negligible response to bovine serum albumin.

In addition, an attempt was made to perform biochemical detections inside the tissue. For example, the catheter, which can provide diagnostic information on the submucosal environment, was reported. Microneedles attached to the catheter tip were proposed to penetrate tissue, detect diagnostic analytes, and perform therapy inside the tissue.^[^ ^138^ ^]^ The microneedles at the catheter tip had a core–shell structure with a micro‐machined shell as a protective layer. The gold core was functionalized for therapeutic and diagnostic purposes to detect metabolites (e.g., glucose, reactive oxygen species (ROS), and uric acid) and ions (e.g., Na^+^, K^+^, Ca^2+^, and pH), which can be important parameters for the diagnosis of bladder disease. Furthermore, the device can help surgeons with functions for bladder surgery such as drug delivery, electroporation, and myoelectric monitoring and modulation. During the in vivo test, the device was combined with an endoscope and entered the narrow urethra of an anesthetized rabbit.

## 4 Sensor‐Integrated Robotic Surgery Tools

Robotic surgery has been developed to overcome the limitations of current minimally‐invasive surgical procedures by enhancing the precision and capabilities of surgeons to levels similar to those seen in open surgery. The first robot surgery was reported as “Arthrobot” developed by the University of British Columbia and the Vancouver General Hospital, which only assisted in changing the patient's leg position controlled by the voice command of the surgeon. Robotic surgery has rapidly expanded beyond its initial use and has become widely used in various fields, including ophthalmology,^[^ ^139^ ^]^ heart surgery,^[^ ^140^ ^]^ thoracic surgery,^[^ ^141^ ^]^ and even pediatric surgery.^[^ ^142^ ^]^ However, precise robot control has been a primary concern in improving the robotic surgery system. The most widely adopted method is feedback control, which can be obtained by physically and chemically monitoring the surgical site. A robotic surgery system mainly comprises two parts: manipulators and end‐effectors. In an analogy to the human arm, manipulators can be compared to arms, while end‐effectors can be compared to hands. In detail, the manipulator moves the end‐effector to the surgical sites, and the end‐effector performs the surgery. Of the two parts, the end‐effector has been the focus of research and development for integrating sensors to monitor the surgical site. This is because the end‐effector is in direct contact with the surgical location and the lesion that needs to be treated. This section presents instances of robotic surgery applications utilizing end‐effectors integrated with sensors, categorized by their applications. The broad usage of sensor‐integrated robotic surgery tools for haptic feedback, especially for medical needles and forceps will first be introduced. Then, more specific usage of sensor‐integrated robotic surgery tools, such as sensor‐driven analysis in medical training, precise tissue handling with subtle force (membrane peeling and cannulation in small vessels), and brachytherapy will be introduced.

### 4.1 Haptic Feedback for Teleoperative Robotic Surgery

#### 4.1.1 Haptic Feedback through Medical Needle at the Robotic End‐Effector

A needle is one of the most frequently utilized medical devices in surgical procedures. The largest field in robotic surgery performed with the needle‐type end‐effector is interventional operation such as biopsy. Almost every sensor‐integrated medical needle introduced in Section 3 can be potentially adopted in this system. However, the needle tip force/pressure is the most commonly monitored for robotic surgery because the physical contact between medical devices and tissues provides the surgeon with the most useful information for various applications.

Integrating various force sensors on the surface of medical needles was technically challenging due to their size, typically smaller than an 18‐gauge needle (corresponding to 1.2 mm in diameter). Therefore, many researchers have tried to monitor the mechanical forces during needle insertion by measuring the transferred mechanical forces at the opposite end of the medical needle. For example, Saito and Togawa installed the mechanical force sensor at the end of the medical needle, and the whole needle and system were mounted on the ultrasonically driven linear stage. Then, they tracked the reaction force during needle insertion through the rabbit ear and successfully confirmed the distinct release of reaction force when the medical needle penetrated the blood vessel. The result could demonstrate the possibilities of autonomous blood sampling and needle placement by tracking applied needle force during insertion.^[^ ^143^ ^]^ Fischer proposed a system with a force sensor to measure the reaction and friction forces during needle insertion, utilizing the optical‐fiber‐based force sensor system. Multiple optical fibers and a spherical mirror with a fixed light source were integrated inside the opposite end of the needle. The position of the spherical mirror varies depending on the reaction and friction forces applied to the needle, resulting in changes in the intensity of the transmitted light through each light fiber. The optical‐fiber‐based force sensor could be integrated with a miniaturized medical needle system using a microscale optical fiber with excellent sensitivity.^[^ ^144^ ^]^ A similar approach for force sensing has been proposed for spinal needles used during lumbar puncture procedures. The design incorporates FBG sensors to measure the compressive force at the needle tip while minimizing the impact of needle bending through structural design.^[^ ^145^ ^]^ Technological advancements have enabled the integration of sensors at the tip of medical needles. Fabry‐Pérot interferometer (FPI)‐based force sensors at the tip of the needle have been demonstrated using three different methods: quartz capillary, Invar capillary, and thin polyimide film. The quartz capillary‐based method exhibited favorable sensing specifications but faced challenges when integrated with small‐diameter needles. On the other hand, the Invar capillary and thin polyimide film‐based methods showed relatively lower sensing specifications but were more feasible for integration in small‐diameter needles.^[^ ^146^ ^]^

**Figure 8:**
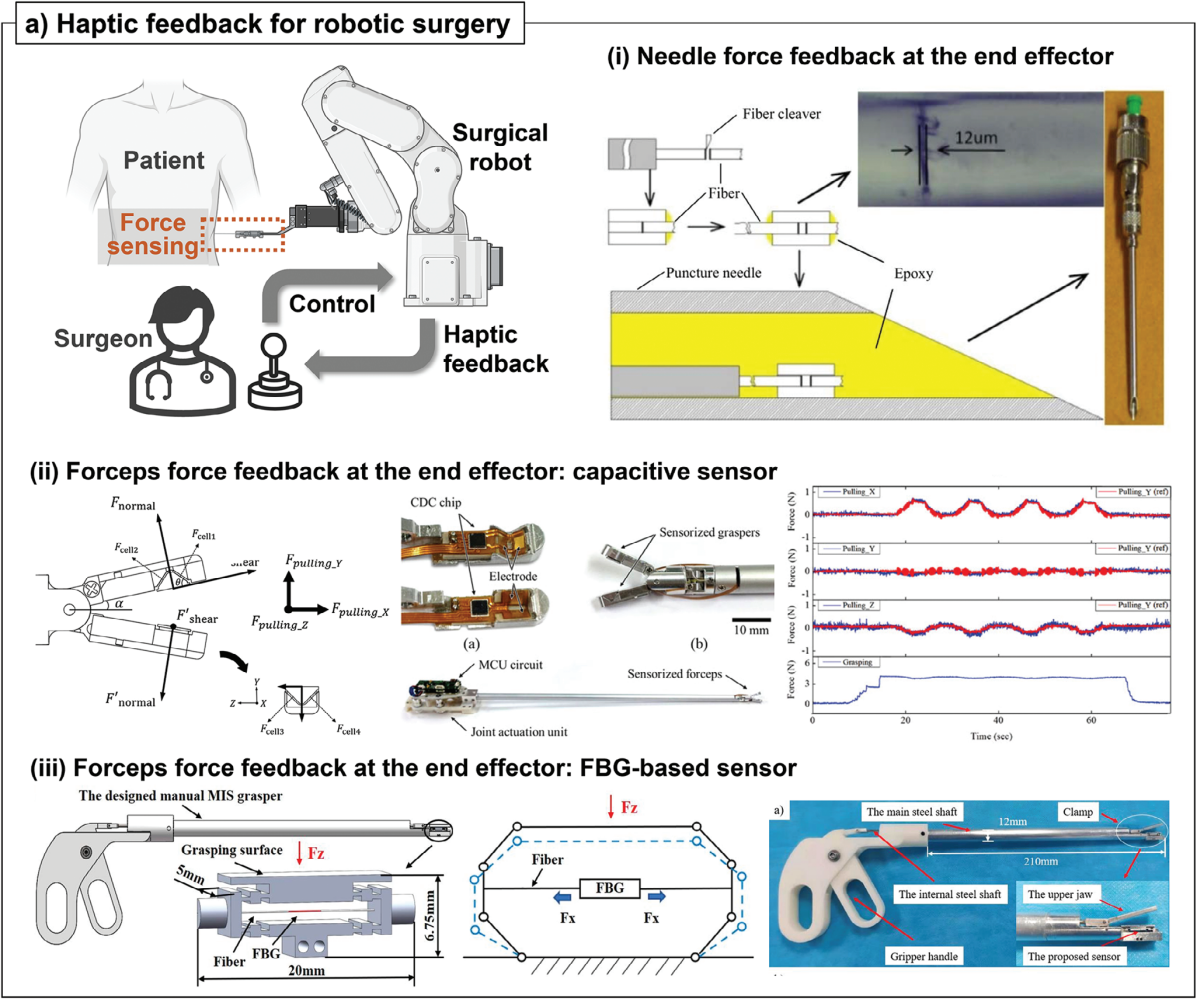
a) Haptic feedback system for teleoperative surgery applications: i) needle end effector with Fabry‐Pérot interferometer force sensor for force feedback system^[^ ^154^ ^]^ (Reprinted with permission.^[^ ^154^ ^]^ Copyright 2015, IEEE), ii‐iii) forceps, end effector used in robotic surgery. Forceps end‐effectors realized with various types of sensors including ii) capacitive type force sensors^[^ ^157^ ^]^ (Reprinted with permission.^[^ ^157^ ^]^ Copyright 2015, IEEE), and iii) FBG‐based sensors^[^ ^159^ ^]^ (Reprinted with permission.^[^ ^159^ ^]^ Copyright 2021, IEEE).

Since minimal invasiveness is the primary purpose of needle‐based surgery, the visualization of the surgical sight is inevitably limited. Therefore, additional monitoring methods should be accompanied by safe needle‐based surgery. MRI is the most frequently adopted method to monitor surgical sites, but it needs high magnetic fields for operation, which can cause an error in sensors composed of metallic components; thus, the development of MR‐compatible sensors is essential for robotic surgery. The first MRI‐compatible sensors adopted are strain gauge‐based force sensors with MR‐compatible materials;^[^ ^147^ ^]^ however, due to the high magnetic noise affecting the measurement of strain gauge‐based force sensors that rely on electrical signals, optical sensors such as FPI have been adopted as an alternative (**Figure** **8**a(i)).^[^ ^148^, ^149^, ^150^, ^151^, ^152^, ^153^, ^154^ ^]^ For example, the MR‐compatible FPI‐based teleoperation system^[^ ^149^, ^151^ ^]^ and signal quality‐improved FPI by Titanium coating^[^ ^150^ ^]^ have been developed.

#### 4.1.2 Haptic Feedback through Medical Forceps at the Robotic End‐Effector

The force feedback loop between the end‐effector and the manipulator is a critical component for robot‐assisted minimally invasive surgery. The lack of force information during a procedure can lead to applying forces that are 1.5 to 2 times higher than intended, potentially causing significant damage to the surgical sites.^[^ ^155^ ^]^ Consequently, significant efforts have been aimed at integrating various sensing modalities into the forceps of end‐effectors in robotic surgery. These forceps are frequently used to manipulate the surgical site and perform the necessary medical procedures. One proposed solution for achieving this is using FBGsensors to monitor forces at the interface between a medical tool and tissue. In the early stage, four FBG sensors were integrated on the joint of forceps instead of being integrated into the forceps to monitor the 3‐degree‐of‐freedom (DOF) force in the x, y, and z‐directions.^[^ ^155^ ^]^ Being advanced from this, H. Li and S. Nakano et al. developed forceps with actuation functions via pneumatic artificial muscle and force monitoring on forceps by observing and modeling disturbances from the external environment.^[^ ^156^ ^]^ However, previous methods could not measure the clamping force, which should be monitored to prevent damage at the clamping region. Instead, previous methods only monitor the force applied at the connecting region between the end‐effector and manipulator. To measure the force applied to overall forceps, a miniaturized capacitive force sensor was integrated at the contact face of the forceps^[^ ^157^ ^]^ (Figure 8a(ii)). This setup enabled the measurement of 4‐DOF forces, which included 3‐DOF pulling forces and 1‐DOF grasping force. Similarly, a biocompatible robotic system for endoscopic surgery was developed using an FBG sensor with tendon‐sheath mechanisms.^[^ ^158^ ^]^ By integrating the FBG sensor at the tendon, the clamping force could be measured directly from the tendon, which is strain‐sensitive^[^ ^159^ ^]^ (Figure 8a(iii)).

### 4.2 Haptic‐Assisted Medical Training

**Figure 9:**
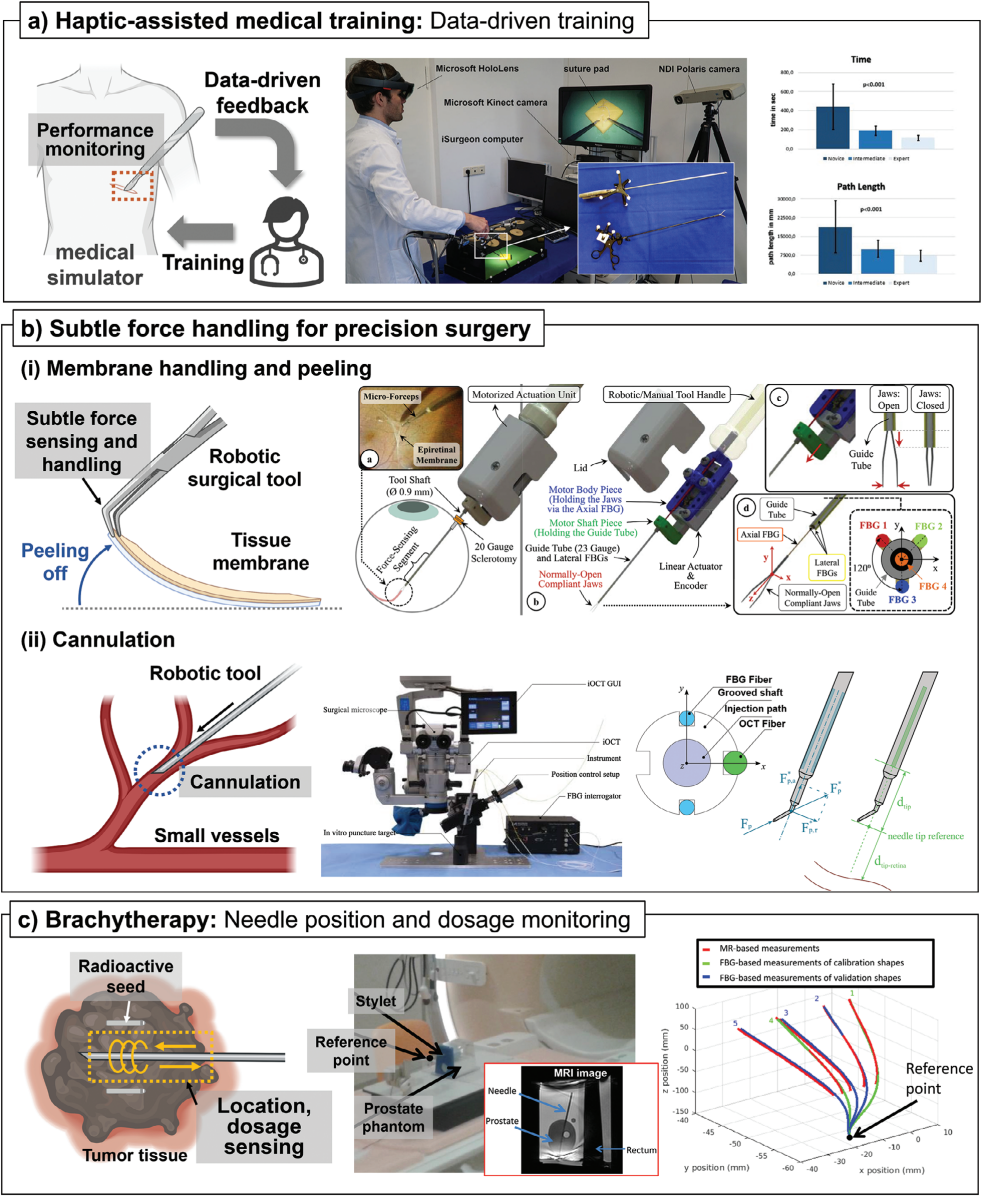
Other sensor‐integrated medical tools for robotic surgery applications. a) Haptic‐assisted medical training providing information and guideline for laparoscopic knots with haptic‐based feedback and evaluation^[^ ^161^ ^]^ (Reprinted with permission.^[^ ^161^ ^]^ Copyright 2016, Springer Science Business Media). b) Subtle force handling for precision surgery: i) a sensor‐integrated end‐effector for membrane peeling in vitreoretinal surgery utilizing FBG‐based extremely sensitive force^[^ ^166^ ^]^ (Reprinted with permission.^[^ ^166^ ^]^ Copyright 2017, IEEE). ii) A sensor‐integrated end‐effector for retinal vein cannulation. The OCT‐A scan module monitoring the exact site of the retinal vein, and two FBG sensors monitoring the puncturing force to assist the cannulation process^[^ ^169^ ^]^ (Reprinted with permission.^[^ ^169^ ^]^ Copyright 2018, IEEE). c) MR‐compatible FBG‐sensor‐integrated needle to estimate the needle tip position^[^ ^171^ ^]^ (Reprinted with permission.^[^ ^171^ ^]^ Copyright 2016, American Association of Physicists in Medicine).

The master‐slave system frequently adopted for teleoperation can be utilized in haptic‐based training systems by replacing the real‐world master component with a simulation. This approach has gained attention because haptic‐based training can be conducted without patients, reducing training costs and potential patient risks that may arise with traditional training methods. Since most surgery has been highly dependent on the surgeon's experience, it is evident that the collection of the reliable and empirical dataset should be preceded to construct the simulation system for haptic‐based training. The needle tip force data were collected during an interventional radiology (IR) with a force sensor‐integrated needle for this application. The force information to puncture the liver, kidney, and muscle was collected, and the required force was summarized to realize haptic feedback during simulation.^[^ ^160^ ^]^ A similar approach was tried in laparoscopic training with the system named iSurgeon. A pre‐established iSurgeon system was used to incorporate empirical data from surgeons to enable haptic‐based training. Data were collected during laparoscopic knot tying using external sensor systems, such as motion‐capturing systems. The results showed noticeable differences in motion patterns between expert, intermediate, and unskilled doctors (**Figure** **9**a).^[^ ^161^ ^]^ Research to set a guideline by quantifying the degree of training has been actively researched using a sensor‐integrated medical system. C. Wu et al. attempted to determine the cognitive and behavioral state of trainees using an engagement index,^[^ ^162^ ^]^ pupil diameter, and gaze entropy,^[^ ^163^ ^]^ which can be calculated by electroencephalogram (EEG) and eye movement. An external sensing system measured both EEG and eye movement. The suggested method could predict the training outcomes with 72.5% accuracy and thus could be adopted as a complementary tool for medical educators and learners during training.^[^ ^162^ ^]^

### 4.3 Subtle Force Handling for Precision Surgery

#### 4.3.1 Membrane Peeling

During vitreoretinal surgery, delaminating ultrathin fibrous tissue from the retina requires the monitoring of highly subtle forces (<10 mN), which are beyond the sensing limit of the human hand.^[^ ^164^ ^]^ This delicate work makes the process challenging, as even a tiny amount of mistakenly applied force could damage the retina. While conventional forceps lack the resolution necessary for this delicate task, sensor‐integrated forceps can assist. Micro‐forceps integrated with force sensors capable of differentiating even subtle forces have been developed to address this issue. For example, an integrated low‐coherence FPI can measure the subtle force (calibration step of 0.897 mN) at medical pick to monitor the delamination of the fibrous tissue.^[^ ^165^ ^]^ Further advancements have demonstrated micro‐forceps that measure the tensile force applied to delaminate tissue. The 4‐DOF force (3 DOF for the tool's x, y, and z‐direction force and 1 DOF for tensile force) was monitored via 4 FBG sensors. The fabricated system shows a force sensing range of 25, 17, and 23.35 mN (estimated from the graph), with RMS error of 0.16, 0.07, and 1.68 mN for *F*_x_, *F*_y_, and *F*_z_, respectively (Figure 9b(i)).^[^ ^166^ ^]^

#### 4.3.2 Cannulation in Small Vessels

The retinal vein is an extremely thin vein located inside the eye, and its occlusion is a common occurrence that can result in vision loss. Retinal vein occlusion is typically treated via retinal vein cannulation, which involves delivering anti‐coagulant drugs to the affected area. However, manually achieving retina vein cannulation can be challenging due to the fragility of the retina vein. Thus, there are considerable demands in retinal vein cannulation via robotic surgery with feedback from integrated sensors. A robotic hand‐held device for retinal vein canulation that can detect the puncture point of the retinal vein by three FBG sensors and automatically hold the position based on the tremor canceling feature has been demonstrated.^[^ ^167^ ^]^ Moreover, distance sensing via optical coherence tomography by single lay (OCT‐A scan) was further incorporated to detect fundamental force and improve accuracy. OCT‐A scan irradiates the laser that selectively reflects the red blood cells and enables the depiction of blood vessels without additional usage of the intravascular dyes. Accompanied by the OCT‐A scan, surgeons can more accurately locate the retinal vein and integrated FBG sensors can assist in monitoring cannulation (Figure 9b(ii)).^[^ ^168^, ^169^ ^]^

### 4.4 Brachytherapy

Brachytherapy is a type of radiotherapy performed by implanting the radioactive seed beside the tissue that needs to be irradiated. One of the advantages of brachytherapy is that it can be performed less invasively, thereby minimizing the impact on the patient. The radioactive seed for brachytherapy is traditionally implanted into the patient's lesion using an inserted needle. However, due to the flexible nature of the needle, the seed can be placed in the wrong location, away from the target site. This error and mislocation of the radioactive seed cause damage to normal tissue with unintentional irradiation. To address this issue, T. Lehmann et al. tried to predict the inserted unseen needle by tracking the force and moment at the needle end. An integrated 4‐DOF force sensor evaluated the force and moment, and the shape of the needle was virtually reconstructed.^[^ ^170^ ^]^ In contrast, an FBG‐sensor‐integrated needle was used during robotic brachytherapy to provide readouts and estimate the needle's shape, and its feasibility in using MR‐compatible FBG sensors was confirmed (Figure 9c).^[^ ^171^ ^]^ As a different approach, a terbium‐doped gadolinium oxysulphide (Gd_2_O_2_S:Tb) was utilized to convert the radiation energy into visible light. An optical fiber and light intensity sensor were used to transport the light and measure the radiation dose. Therefore, radiation dose could be successfully monitored by measuring the light intensity converted from the radiational energy.^[^ ^172^, ^173^ ^]^ When two methods are combined, more accurate brachytherapy can be performed. For example, an electromagnetic position sensor and radiation dose sensor with a plastic scintillation dosimeter were fused, significantly improving accuracy.^[^ ^174^ ^]^

## 5 Other Examples of Sensor‐Integrated Medical Tools and Their Applications

This chapter discusses sensor‐integrated medical devices that were not categorized in previous chapters. Specifically, we will discuss sensor‐integrated medical tools such as sutures, endoscopes, and intubation tubes and those used in conjunction with medical imaging devices, such as MRI and electrical impedance topology (EIT).

### 5.1 Suture

A suture is a thin thread or material used to seal wounds or join tissues after surgery.^[^ ^59^ ^]^ Because the suture should be able to hold tissues stably during the wound healing process, various material properties such as tensile strength, knot security, tissue reactivity, and wound security are often required.^[^ ^179^ ^]^ After suturing, monitoring the suture site during wound healing and rehabilitation is complex without additional incisions and invasive techniques. Therefore, a cautious approach with extended recovery time and a large security margin has been employed to ensure complete recovery. For this reason, sensor integration inside sutures to monitor wounds after suturing provides the benefit of real‐time monitoring of an individual's wound site.

**Figure 10:**
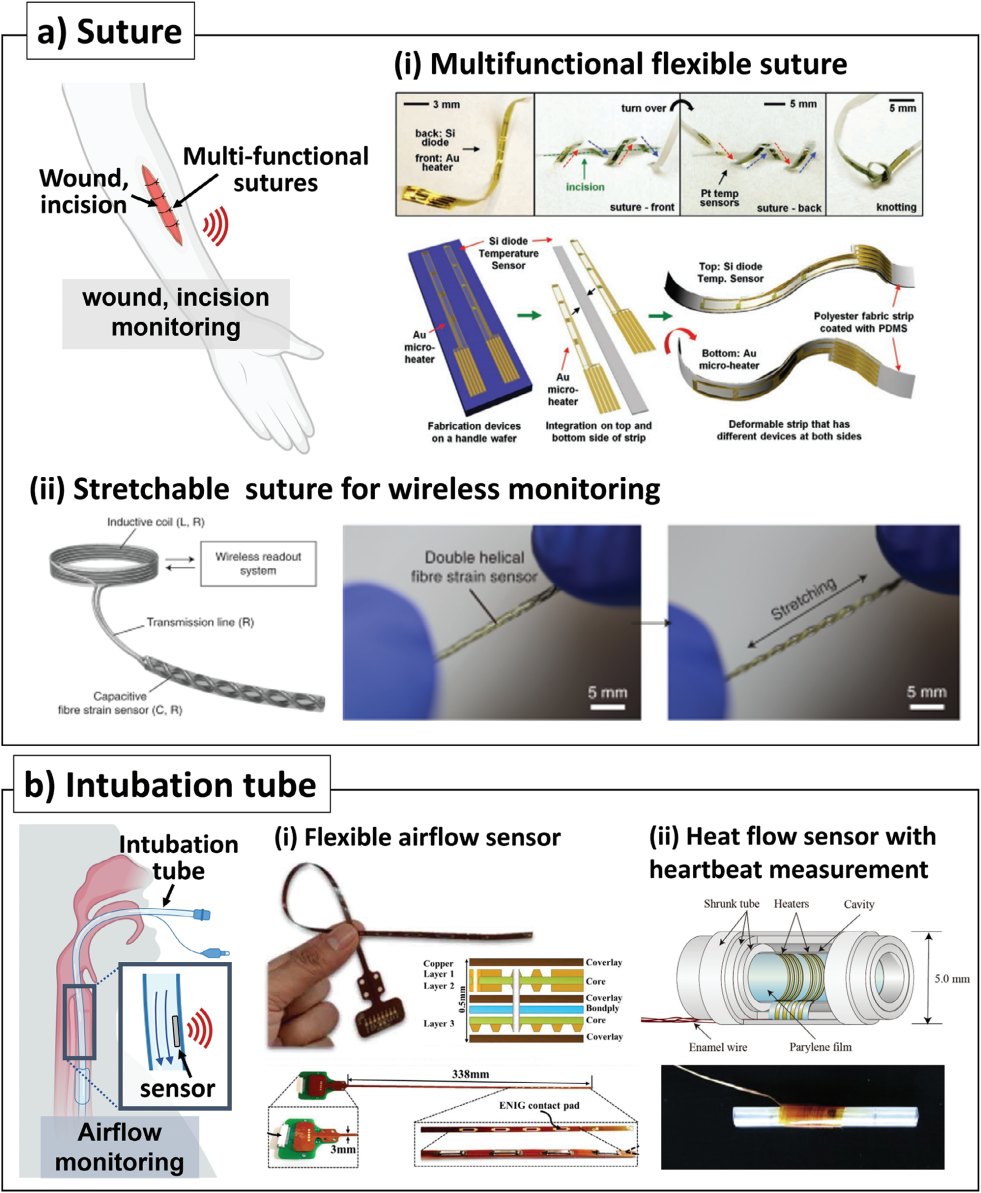
Other sensor‐integrated medical tools. Sensor‐integrated a) suture: i) sutures with temperature sensors and an Au heater^[^ ^175^ ^]^ (Reproduced with permission.^[^ ^175^ ^]^ Copyright 2012, Wiley‐VCH) and ii) suture for continuous monitoring with wireless communication^[^ ^176^ ^]^ (Reprinted with permission.^[^ ^176^ ^]^ Copyright 2021, The Author(s), under exclusive license to Springer Nature Limited), b) intubation tube: i) steerable intubation catheter for airflow measurement^[^ ^177^ ^]^ (Reprinted with permission.^[^ ^177^ ^]^ Copyright 2021, IOP Publishing, Ltd), and ii) cardiac and respiratory signal measurement with the endotracheal intubation tube^[^ ^178^ ^]^ (top figure)(Reprinted with permission.^[^ ^178^ ^]^ Copyright 2017, IEEE); and temperature compensated catheter flow sensor^[^ ^207^ ^]^ (bottom figure) (reprinted with permission.^[^ ^207^ ^]^ Copyright 2013, Elsevier).

For example, a suture with capabilities of temperature monitoring and thermal heating to monitor and treat inflammation during wound healing has been proposed (**Figure** **10**a(i)).^[^ ^175^ ^]^ Because the inflammation generally accompanies a local increase in temperature, it could be monitored through the Si nanomembrane diode and Pt resistor‐based temperature sensor. The thermal heater was fabricated for therapeutic function using microfabrication and transfer printing for integration into a small suture strip. Also, the Pt‐coated Au micro‐heater for local Joule heating was used for a therapeutic function. The device was coated and insulated with PDMS and epoxy to adjust the neutral plane to be positioned at the PDMS strain isolation layer. As a result, the suture could endure tensile stress applied during the suturing and was flexible enough to be twisted and knotted with a meander length (wavelength) of 30 mm. During the in vivo mouse model test, the sensor‐integrated suture was placed on a silk substrate for threading and stitching. The sensor‐integrated suture could continue to measure the temperature within the body after the wound was closed with the suture.

Tendon and ligament (T/L) injury can cause abnormal joint function and degenerative disease.^[^ ^180^, ^181^ ^]^ In cases of T/L injury, surgery, and rehabilitation are typically employed to restore the affected tissues to their pre‐injury condition. The biomechanical properties of the tissue strain may provide continuous tissue monitoring during the healing and rehabilitation stage in individuals.^[^ ^182^ ^]^ In light of this, an implantable and biodegradable pressure sensor was proposed to monitor tendon or ligament conditions before and after injuries.^[^ ^183^ ^]^ Likewise, a strain sensor in a suture form was also reported, which can wirelessly monitor T/L damage (Figure 10a(ii)).^[^ ^176^ ^]^ In this work, the suture comprised liquid‐metal‐based fibrous electrodes for capacitive strain sensing and an inductive coil for wireless tissue monitoring. Two stretchable conductive fibers were twisted into a helical shape for capacitive sensing and then connected to an inductor coil for wireless readout. Ex vivo and in vivo studies with a porcine leg demonstrated its possibilities for orthopedic application. Multi‐filament surgical sutures with the pledgets for RF identification were reported.^[^ ^184^ ^]^ They were made of conductive polymer, poly(3,4‑ethylene dioxythiophene)‐poly(styrene sulfonate) (PEDOT:PSS), and the pledget was connected to suture for a battery‐free measurement of harmonic backscattering spectra, which significantly reduced the form factor of the functional suture system. In vivo test with a porcine model demonstrated the deep‐wound sensing capacity of the proposed system, with the ability to monitor the condition of stitched sutures without requiring direct access to the surgical site. Additionally, the system enabled monitoring of the wound healing process.

### 5.2 Intubation Tube

Severe constriction of the central airway requires careful monitoring, timely diagnosis, and management. Detection of narrowing airways in pediatric patients is vital due to small anatomical geometry. A slight reduction in cross‐section area can exponentially increase the resistance to flow for the pediatric population. To determine airflow patterns in the stenosed trachea, it is necessary to monitor air velocity within the tracheobronchial tree using an intubation tube. Therefore, an intubation tube with a MEMS heat flow sensor and helical shape memory actuators (SMA) has been reported (Figure 10b(i)).^[^ ^177^ ^]^ The heat flow sensor in the intubation tube was fabricated with MEMS fabrication of n‐doped silicon, which detected the airflow inside the tracheobronchial tree, while the SMA placed on a distal end of the tube was adopted to steer the tube tip. An increased air flow rate with decreasing lumen cross‐sectional area was observed using excised sheep tracheal tissue. In addition to measuring breathing signals, an endotracheal intubation tube was also used to detect cardiac signals simultaneously with respiratory data (Figure 10b(ii)).^[^ ^178^ ^]^ The heat flow through the tube was converted into a respiratory and cardiac rhythm, and the periodic peak from the flow sensor coincided with a conventional electrocardiogram. The heat flow sensor was made with microfabrication on polyimide so that it could be rolled and attached around the surface of the intubation tube. With flexible, miniaturized sensors attached to the surface of the intubation tube, it realized in vivo measurement of the respiratory signal from a rat. The device measured not only respiratory signs but also cardiac signals, which are another essential factor for monitoring and diagnosing patients’ conditions.

### 5.3 Multifunctional Endoscopes

An endoscope is a medical inspection device to examine organs inside the body, and it is commonly inserted through the gastrointestinal tract. It comprises a flexible tube, camera, lens, and illumination system, allowing optical inspection inside the body. However, beyond visual observation, analyzing lesions in various aspects, such as biophysical and biochemical parameters, can significantly enhance the examination quality and perform treatment and diagnosis in real‐time. Therefore, multiple efforts to integrate sensor functionalities into the endoscope have been made and reported.

An endoscope with a miniaturized ion‐selective sensor array for pH and potassium ion detection was proposed to analyze local microvascular perfusion and tissue oxygenation to prevent ischemia.^[^ ^190^ ^]^ It is a well‐established fact that during ischemia, anaerobic respiration results in a decreased production of adenosine triphosphate (ATP), which negatively impacts the sodium‐potassium pump and causes an accumulation of potassium ions in the extracellular fluid.^[^ ^191^ ^]^ Residual carbon dioxide in the bloodstream triggers a reaction that produces hydrogen and bicarbonate ions, decreasing pH levels.^[^ ^192^ ^]^ Therefore, potassium and pH detection electrodes were combined at the proximal end of the natural orifice transluminal endoscopy. Specifically, ion‐selective working electrodes were decorated with potassium or hydrogen ionophores and fixed at the tip of the endoscope for potassium and pH‐selective detection. Reference electrodes were coated with Ag/AgCl and Nafion for stable measurement in a biochemical environment. The sensor array was tested in vivo on pigs to monitor ischemia resulting from the clamping of blood vessels to restrict blood flow to the stomach. The sensor array was also utilized to inspect the gastric mucosa under highly acidic and corrosive conditions. The study revealed a decrease in potassium concentration and acidic pH levels compared to normal conditions during ischemia, which can be attributed to the effects of anaerobic respiration.

**Figure 11:**
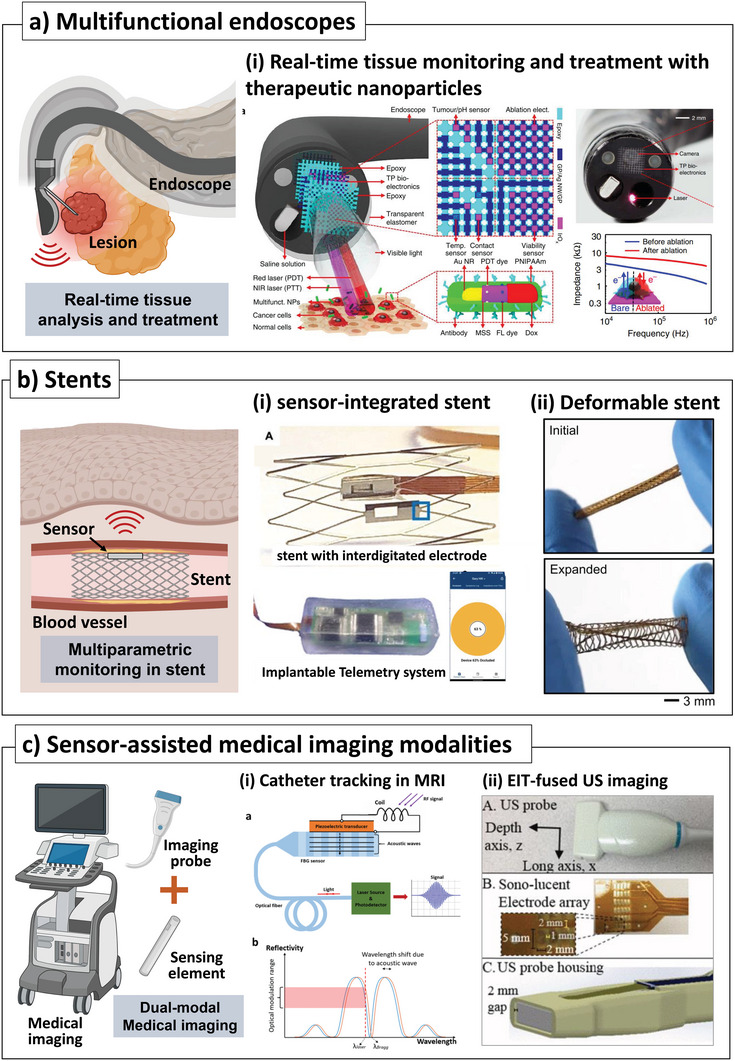
Other sensor‐integrated medical tools. Sensor‐integrated a) multifunctional endoscopes: i) multifunctional surgical endoscope for diagnosing and treating intestinal diseases^[^ ^185^ ^]^ (Reproduced under the terms of the CC‐BY‐4.0 license.,^[^ ^185^ ^]^ Copyright 2015, Springer Nature), b) stents with sensors: i) self‐reporting device that detects the blockage of the cardiovascular stent^[^ ^186^ ^]^ (Reproduced under the terms of the CC‐BY 4.0 license.,^[^ ^186^ ^]^ Copyright 2022, Wiley) and ii) vascular catheter with soft sensors and fully implanted, wireless, and battery‐free stent for multiplex hemodynamic sensing^[^ ^187^ ^]^ (Reproduced under the terms of the CC‐BY 4.0 license.,^[^ ^187^ ^]^ Copyright 2022, Elsevier), and c) sensor‐assisted medical imaging modalities: i) FBG with a piezoelectric sensor for monitoring and guidance of catheters^[^ ^206^ ^]^ (Reproduced with permission.^[^ ^206^ ^]^ Copyright 2019, IEEE) and ii) EIT used with the ultrasonography (US) to measure electrical impedance myography (EIM) to differentiate between healthy and diseased tissues^[^ ^189^ ^]^ (Reprinted with permission.^[^ ^189^ ^]^ Copyright 2019, IEEE).

With the advancements in nanoparticle technology, a sensor‐integrated endoscope, together with nanoparticle technology, enabled in situ monitoring and treatment of tumors inside the tissue with a multifunctional endoscope (**Figure** **11**a).^[^ ^185^ ^]^ The multifunctional endoscope confirmed the presence of tumor‐binding nanoparticles in the mouse body and performed nanoparticle‐assisted thermal and chemo‐therapy with irradiation during the endoscope‐assisted surgery. The endoscope was equipped with multiple functionalities, in vivo biophysical (e.g., temperature, fluorescence‐based mapping), biochemical analysis (e.g., pH, impedance), and laser‐assisted ablation (red, near‐infrared) for tumor treatment. The multiple devices on the endoscope tip were made transparent. Hence, the endoscope still performs optical imaging with multiple functionalities. The endoscope can coincide with nanoparticles placed in a mouse body and diagnose and treat intestinal diseases. Overall, the multifunctional endoscope facilitated on‐site mapping of tumor locations and identification of cancer through pH and impedance measurements before and after treatment. Also, controlled drug release and imaging were possible with a single endoscopic surgery procedure thanks to the targeted delivery of nanoparticles.

### 5.4 Stents

A stent is a tubular medical device made of plastic or springy metal mesh and is used to treat and prevent occlusion in a blood artery (angioplasty) or to assist the recovery after an anastomosis.^[^ ^193^ ^]^ Monitoring the environment surrounding the stent is crucial to prevent potential side effects or disease recurrence. This necessity has increased interest in stents with integrated sensing capabilities. For example, a stent with a passive array of magnetoelastic resonators, which can be potentially used for wireless monitoring of biliary sludge, was reported and utilized to detect a sludge in a bile duct.^[^ ^194^ ^]^ Biliary sludge, a bacterial matrix, typically forms around and on biliary stents, which can lead to the re‐occlusion or infection of the bile duct. Three magnetoelastic ribbon‐shaped sensors (1 mm wide × 25 µm thick) are attached to the inner wall of a stent to monitor the formation of the biliary sludge. Each sensor array was equipped with its designated frequency band, which is tuned with the length of each sensor. The magnitude and phase of the resonator were measured during an in vivo test with swine bile, which showed no adverse effects on the animal's health. A stent electrode array was designed to chronically record cortical neural activity through endovascular means.^[^ ^195^ ^]^ 750 µm platinum disc electrode was welded to a platinum–tungsten wire and attached to a Nitinol‐based stent. The stent electrode array measured somatosensory evoked potential upon stimulation in sheep forelimbs. Also, brain recordings were successfully carried out for up to 190 days, and the vascular electrocorticography obtained with the stent was comparable to that obtained from epidural surface arrays. Similarly, a stent with an electrode array for electrochemical impedance spectroscopy (EIS) measurements was also demonstrated to measure changes at the electrode‐tissue interface.^[^ ^196^ ^]^

After stent deployment, stents are often blocked due to a wound response called stent restenosis.^[^ ^197^ ^]^ To address this issue, a self‐reporting device that detects the blockage of the cardiovascular stent (Figure 11b(i)) was reported.^[^ ^186^ ^]^ The Nitinol stent was custom‐designed with interdigitated Pt electrodes mounted on it, and an implantable wireless transceiver was connected to monitor cell growth through EIS. A wireless, battery‐free communication system with soft sensors was proposed for multiplex hemodynamic sensing applications in stents (Figure 11b(ii)).^[^ ^187^ ^]^ The stent was composed of an inductive antenna with a conductive component and a nonconductive polyimide structure. The stent, integrated with soft capacitive sensors, could monitor vascular conditions by measuring pressure, heart rate, and blood flow. The measured parameters can be wirelessly transmitted via a resonant LC circuit from the stent to an external receiver up to 3.5 cm within the blood. With a rabbit model, the stent integrated with sensors was validated in a silicone artery phantom under pulsatile flow conditions and ex vivo.

Recently, a stent with versatile functionality with advanced materials has also been reported. For example, stretchable electronics are fused with the stent to enable versatile sensing and treatment functionalities.^[^ ^198^ ^]^ Likewise, a stent with bioresorbable materials is actively utilized to reduce additional surgery or avoid stent restenosis.^[^ ^199^ ^]^

### 5.5 Medical Imaging Fused with Sensor Modalities

MRI is often limited in its tracking techniques compared to guiding and tracking devices used in X‐ray fluoroscopy. Therefore, additional components are commonly incorporated to visualize the medical tool under MRI imaging. For example, an acousto‐optic catheter with an FBG and piezoelectric sensor^[^ ^188^, ^206^ ^]^ (Figure 11c(i)) was utilized to track catheter position during MRI imaging. The piezoelectric transducer connected with a tiny coil could convert the RF signal into an acoustic wave in the optical fiber. Then, the acoustic wave detected by a photodetector could be transformed into the position of the tracking coil.

Transrectal ultrasonography (TRUS) is a widely accepted and highly accurate method for identifying and staging prostate cancer during prostate biopsy. However, TRUS has limited accuracy, leading to false‐negative or false‐positive results because malignant tissue can appear as both the hypo‐echoic and hyper‐echoic zones in the ultrasound image.^[^ ^200^ ^]^ Therefore, the transrectal electrical impedance tomography (TREIT) system was proposed as an alternative imaging method.^[^ ^201^ ^]^ The TREIT probe was equipped with electrodes for electrical impedance tomography (EIT) and a conventional ultrasound imaging probe. The TREIT allows for more precise imaging of tissue structure by providing additional structural information. It can provide dimensions, radial distances, angular orientations, and heights relative to the probe axis, which are difficult to be identified by the traditional TRUS method. Similarly, a sonolucent electrode array wrapped around the active face of the ultrasound probe (Figure 11c(ii)) was proposed for electrical impedance myography (EIM).^[^ ^189^ ^]^ The device measured electrical impedance myography (EIM) to distinguish healthy and diseased tissue for localized diagnosis of muscle health.

## 6 Conclusion

Over the past two decades, substantial research has been conducted to incorporate diverse sensing modalities into medical tools. These advances and efforts have enabled safer and more accurate medical treatments by providing useful information to both surgeons and patients. This review introduced the recent advancements and examples of sensor‐integrated medical and surgical tools, and their usefulness on the clinical side was thoroughly discussed. In the case of medical needles and catheters, incorporating various biosensors could enable more accurate placement inside the patient's body by analyzing the types of tissue. Furthermore, integrating versatile biosensors can introduce novel opportunities for the real‐time monitoring of deep tissue and internal bodily lumens. The information collected by integrated sensors can assist clinicians in making the correct diagnosis and performing accurate treatment, resulting in less harm to healthy tissues, less trauma to patients, and a shorter recovery period. For robot‐assisted surgery, integrating physical and chemical sensors within the medical robotic can potentially deliver real‐time and immersive sensory feedback during medical procedures. This advancement has the potential to significantly enhance the quality and overall outcome of medical telesurgery. As introduced in the review, sensor fusions can be extended toward other medical and surgical tools, for example, sutures, tubes, stents, and endoscopes. The sensor fusion realizes short‐term and long‐term monitoring of health‐related information to deal with pre‐/post‐operative complications. As outlined in this review, sensor technology, fabrication techniques, and materials engineering advances have enabled the integration of sensors into medical devices. Initially, sensor integration with medical devices was often achieved by separately mounting bulky sensor components onto medical devices. However, with the help of micro‐fabrication technology, sensor components have been miniaturized, allowing them to be integrated into small medical devices. In addition, recent innovations in flexible and soft sensor technology facilitate the advanced sensor packaging and integration process.^[^ ^35^, ^176^, ^202^ ^]^ Moreover, advanced material design enables more functional medical devices, such as biodegradable implants eliminating additional surgery for removals from the body.^[^ ^203^, ^204^, ^205^ ^]^

However, various limitations and hurdles remain in adopting sensor‐integrated medical tools. First, only a limited number of biophysical and biochemical parameters can be analyzed by state‐of‐the‐art sensor technologies. Most bio‐assay methods highly rely on bulky and bench‐top instruments, especially for detecting bio‐analytes (e.g., metabolite, protein, hormone, and nucleotides). To realize on‐site and in vivo bio‐assay during medical procedures, bio‐assay methods with versatile sensing capabilities in miniaturized and compact systems should be developed. In addition, it is essential to note that the limited reliability and accuracy of biophysical and biochemical sensors impose limitations on their application in the medical field. Presumably, this is why only a few robust transducers, such as load cells, FBG‐type sensors, and electrical impedance sensors, are often adopted for sensor‐integrated medical tools. Last, the required interfaces, such as electronics, mechanical components, and optical systems, must be miniaturized and diversified to incorporate sensor systems into current medical and surgical equipment.

Recent medical cases have demonstrated a notable shift toward adopting minimally invasive approaches in medical procedures. This trend offers significant advantages, including enhanced accessibility of surgical tools to the targeted surgical sites and reduced patient recovery time. Furthermore, the remarkable progress in biomedical and clinical domains has rendered previously impossible treatment cases feasible, increasing demand for such procedures. This trend signifies a greater likelihood of indirect access to surgical sites using various medical devices, thereby resulting in surgeons experiencing a loss of sensory feedback during the procedure. Consequently, there is a rising need for systems capable of analyzing and conveying the physical and chemical information between the surgical tool and the lesion through biophysical and chemical sensors. Moreover, the emergence of artificial intelligence technologies has empowered us to comprehend the intricate relationships between individual biosignals and underlying diseases in patients. This comprehension suggests a growing abundance of opportunities and extensive demands for high‐quality health‐related information in the clinical field. Hence, fostering interdisciplinary approaches and promoting active communication between the engineering and clinical fields will be the bedrock for driving breakthroughs. This collaborative effort enables overcoming the current limitations and the sharing engineering solutions, leading to innovative advancements in healthcare and medical technologies.

## Conflict of Interest

The authors declare no conflict of interest.
